# Allicin inhibits PD-L1 through the IL-6/JAK2/STAT3 pathway to suppress immune evasion in osteosarcoma

**DOI:** 10.3389/fimmu.2026.1735090

**Published:** 2026-02-20

**Authors:** Rui Gong, Xi-min Jin, Xu Cui, Jia-hao Sun, Wen-peng Xie, Yong-kui Zhang

**Affiliations:** 1 First Clinical College, Shandong University of Traditional Chinese Medicine, Jinan, China; 2 Department of Orthopedic Surgery, Affiliated Hospital of Shandong University of Traditional Chinese Medicine, Jinan, Shandong, China

**Keywords:** allicin, IL-6/JAK2/STAT3, immune evasion, osteosarcoma, PD-L1

## Abstract

**Objective:**

PD-L1 is one of the most critical immune checkpoint proteins, inhibiting T-cell immune responses by binding to PD-1. This study aims to validate that allicin can regulate PD-L1 expression through the IL-6/JAK2/STAT3 pathway, thereby inhibiting immune evasion in osteosarcoma.

**Methods:**

We screened differentially expressed genes associated with prognosis using the GEO database and identified the IL-6/JAK2/STAT3/PDL1 pathway through KEGG and GO enrichment analysis. We established the HOS human osteosarcoma cell line and the K7M2 mouse osteosarcoma cell line. Both cell lines were treated with allicin at concentrations of 12.5, 25, and 50 μmol/L. Transwell, clonogenic, and scratch assays validated allicin’s inhibitory effects on osteosarcoma cell growth, migration, and invasion. Western Blot assays measured expression levels of key proteins including IL-6, JAK2, STAT3, PD-L1, and phosphorylated JAK2/STAT3. Animal models were established in Balb/c mice and treated with allicin. Mouse clinical signs, tumor volume, and size were recorded. Tumor microenvironment and immune cell infiltration markers (CD3+, CD4+, CD8+, IFN-γ, granzyme B) were analyzed via flow cytometry and immunofluorescence. Immunofluorescence and immunohistochemistry were employed to detect the expression of PD-L1, CD8, and other relevant markers in mouse tumor models, validating allicin’s inhibitory effect on immune evasion.

**Results:**

In osteosarcoma cell lines treated with allicin, the IL-6/JAK2/STAT3 signaling pathway was downregulated, and PD-L1 expression was significantly suppressed. In allicin-treated mice, recruitment of CD4+ and CD8+ T cells increased, IFN-γ and granzyme B expression enhanced, and tumor immune evasion was markedly inhibited.

**Conclusion:**

Allicin suppresses PD-L1 expression by modulating the IL-6/JAK2/STAT3 signaling pathway, thereby improving the tumor microenvironment and inhibiting immune evasion in osteosarcoma cells. This study demonstrates the potential of allicin as an adjunct to immunotherapy.

## 1 Introduction

Osteosarcoma is a highly heterogeneous tumor originating from mesenchymal tissue, characterized by high invasiveness and primarily occurring in children and young adults (1). Approximately 3 to 4.5 new cases per million population annually (2). With the continuous advancement and optimization of treatment protocols, 60% of patients with localized osteosarcoma achieve a 5-year survival rate. However, for patients with metastatic or recurrent disease, the 5-year survival rate is only 20% (3). The high mortality and disability rates impose a heavy social and economic burden, making the development of new treatments particularly crucial.

The immune system can detect and eliminate diseased or potentially harmful cells. Unfortunately, tumors can evade the immune system through various mechanisms, such as reducing antigen presentation, blocking the initiation of T cell immune responses, or suppressing T cell effector functions (4). Immune checkpoint blockades have achieved tremendous success across multiple tumor types. They work by blocking inhibitory signals that suppress T cell activation, thereby enabling more CD8+ T cells to become activated and kill tumor cells (5). The PD1/PD-L1 axis is one of the primary immune checkpoints and is also considered a major driver of tumor immune evasion. Tumors utilize their own PD-L1 (CD274) to bind with PD-1 on T cells, thereby blocking the immune system’s ability to kill them (6). Consequently, inhibiting PD-L1 to block tumor immune escape represents a highly promising therapeutic approach. Previous studies have demonstrated that elevated PD-L1 expression correlates with poor prognosis in osteosarcoma patients. A research team demonstrated that quercetin inhibits PD-L1, producing significant therapeutic effects against osteosarcoma *in vitro* experiments (7). However, current immunotherapy targeting the PD-1/PD-L1 axis for osteosarcoma has shown suboptimal efficacy when used as monotherapy (8, 9). There remains a need to develop suitable drugs for combination therapy.

Allicin (diallyl thiosulfate) is an organic sulfur compound with multiple biological activities (10). Allicin exhibits inhibitory effects on various tumors including liver cancer, colorectal cancer, and melanoma (11–13). Our previous research has also demonstrated that allicin can treat osteosarcoma through multiple mechanisms (14). Allicin exerts antibacterial effects in infectious diseases, is regarded as a natural antibiotic, and simultaneously modulates the body’s immune response (15). In our previous research, allicin was found to exert a regulatory effect on the immune microenvironment of osteosarcoma. This finding is consistent with the results of a recent study (16). However, the effects of allicin on PD-L1 and immune evasion remain unknown at present.

In this study, we demonstrated that allicin inhibits PD-L1 expression in osteosarcoma cells and elucidated that this effect is mediated through regulation of the IL-6/JAK2/STAT3 pathway. In mouse tumor models, we confirmed allicin’s suppression of osteosarcoma immune evasion and its regulatory impact on the tumor microenvironment. Collectively, these findings indicate that allicin holds potential for clinical application in osteosarcoma immunotherapy.

## 2 Materials and methods

### 2.1 Cell culture

The 143B (CRL-8303), HOS (CRL-1543), MG63 (CL-0157), U2OS (CL-0236) and K7M2-WT (CL-0371) cell lines were obtained from Procell Life Science & Technology Co., Ltd., China. The hFOB 1.19 (GNHu14) cells were obtained from the Cell Bank of Chinese Academy of Sciences Type Culture Collection (CBTCCCAS). All cell lines were identified by STR analysis. The K7M2-WT, MG63 and U2OS cell lines were cultured in Dulbecco’s Modified Eagle Medium (DMEM) (Procell Life Science & Technology). The 143B and HOS cell line was cultured in Minimum Essential Medium (MEM) (Procell Life Science & Technology). All media listed above were supplemented with 10% fetal bovine serum (FBS) (Procell Life Science & Technology) and 1% penicillin-streptomycin (Procell Life Science & Technology). The hFOB 1.19 cells are cultured in dedicated complete medium (CM-0353, Procell Life Science & Technology). All cells were maintained at 37 °C in 5% CO2 humidified atmospheres.

### 2.2 Allicin intervention in cells and rescue experiments

Allicin (M9963, AbMole, shanghai, China, >98%) was dissolved in DMSO. Solutions of allicin at concentrations of 12.5, 25, and 50 μmol/L were prepared using the DMSO solution, with the final DMSO volume fraction set at 0.1%. Add allicin solution to the culture medium of HOS and K7M2 cells. At the same time, establish untreated HOS and K7M2 cell lines as control groups under the same culture conditions.

Colivelin (STAT3 activator, MedChemExpress, New Jersey, USA) was dissolved in DMSO. A solution containing 50 μg/mL was prepared using DMSO, ensuring the DMSO volume remained below 2%. Seed HOS and K7M2 cells into 10cm cell culture dishes, after reaching 70%–80% confluence, treat the cell lines with allicin for 24 hours, then treated with Colivelin for 4 hours.

### 2.3 RNA extraction and real-time quantitative polymerase chain reaction analysis

Total RNA was extracted from cells treated with allicin using the RNA-Quick Purification Kit (RN001, Yishan, China). Following the kit instructions to set reaction parameters, GAPDH was used as an internal control. PD-L1 expression levels in each cell line were quantified using the 2−ΔΔCt method. Primer sequences are listed in the **Table 1** below.

**Table 1:** The primers sequences of genes.

Name of the encoded protein	Forward primer (5′-3′)	Reverse primer (5′-3′)
PD-L1	GGTAAGACCACCACCACCAAT	TGATTCTCAGTGTGCTGGTCAC
GAPDH	GCACCGTCAAGGCTGAGAAC	TGGTGAAGACGCCAGTGGA

### 2.4 CCK-8 assay

Cell Counting Kit-8 (G4103) was purchased from Servicebio (Wuhan, China). Prepare 1.5× 10^3^ HOS and K7M2 cells in a 100 μL cell suspension and seed them into a 96-well plate. After 24 hours, once the cells have fully adhered. Both cell lines were treated with allicin at concentrations of 12.5 μmol/L, 25 μmol/L, 50 μmol/L, 100 μmol/L, and 200 μmol/L for 24 and 48 hours. Add 10 μL of CCK-8 to each well and incubate for 2 hours. Measure the optical density (OD) at 450 nm using a microplate reader (Thermo Fisher).

### 2.5 Colony formation assay

Each cell group was seeded at a density of 3 × 10^3^ cells per well in a 6-well plate and cultured for 14 days at 37 °C with 5% CO_2_, with medium changes every two days. Cells were fixed with 4% paraformaldehyde and stained with crystal violet, followed by colony counting using ImageJ software.

### 2.6 Scratch wound assay

HOS and K7M2 cells were seeded into 6-well plates at a density of 3 × 10^5^ cells per well. Once cells have confluently seeded, scrape the cell layer using a 200 μL pipette tip. After thoroughly washing away the scraped cells, apply allicin treatment. Immediately observe cells under a microscope following the scratch, then re-observe after 24 hours and record cell migration status.

### 2.7 Transwell assays

Transwell chamber (3422) was supplied by Corning (New York, USA). After 24 hours of allicin treatment, resuspend 1 × 10^5^ cells in 200 μl of medium. For the migration assay, seed cells into the upper transwell chamber of serum-free medium. For invasion assays, Matrigel (Corning) was first coated onto the upper transwell chamber. Add medium containing 20% FBS to the lower transwell chamber. After 24 hours of culture, remove the upper transwell chamber and scrape off cells adhering to the surface using a cotton swab. Fix cells on the lower surface with 4% paraformaldehyde at room temperature for 30 minutes, stain with crystal violet solution (CB0331, Sangon Biotech, Shanghai, China), and observe under a microscope.

### 2.8 Western blot

RIPA lysis buffer (Solarbio), PMSF (Solarbio), and phosphatase inhibitor (Solarbio) were mixed at a ratio of 1 mL:10 μL:10 μL to prepare the protein lysis buffer. After collecting the cleaved proteins, perform protein quantification using the BCA kit (Solarbio). Protein samples were separated by SDS-polyacrylamide gel electrophoresis (SDS-PAGE) and transferred to PVDF membranes. The membrane was blocked with 5% milk at room temperature for 1 hour, followed by overnight incubation with primary antibodies at 4 °C. The primary antibodies used were as follows: PD-L1 (Proteintech, 66248-1-IG, China), IL-6 (Proteintech, 21865-1-AP, China), STAT3 (Affinity, AF6294, China), Phospho-STAT3 (Affinity, AF3293, China), JAK2 (Affinity, AF6022, China), Phospho-JAK2 (Affinity, AF3024, China), and GAPDH (Affinity, AF7021, China). Incubate with secondary antibody at room temperature for 1 hour. Protein bands were visualized using a highly sensitive chemiluminescent reagent and analyzed with ImageJ.

### 2.9 Tumor allografts model

Animal experiments were approved by the Institutional Animal Care and Use Committee and Animal Ethics Committee of Affiliated Hospital of Shandong University of Traditional Chinese Medicine (approval number SDSZYYAWE20250615001). Twelve four-week-old male BALB/c mice were purchased from Jiangsu Huachuang Xinnuo Pharmaceutical Technology Co., Ltd. (Jiangsu, China). They were housed under specific pathogen-free (SPF) conditions at the Animal Experiment Center of the Affiliated Hospital of Shandong University of Traditional Chinese Medicine. A 5×10^6^ K7M2 cell injection was administered into the left axilla of each mouse. When the neoplastic size was grown to about 0.05 cm^3^, mice were divided into two groups: the control group received an intraperitoneal injection of saline, while the treatment group received an intraperitoneal injection of allicin (50 mg/kg), with 6 mice per group. Body weight and tumor volume were recorded throughout the study period. After 14 days of treatment, mice were euthanized, and the allograft tumors were excised. Tumors were either paraffin-embedded or prepared as a single-cell suspension.

### 2.10 Immunofluorescence and immunohistochemistry

Paraffin-embedded tumor sections were processed using Environmental Friendly transparent dewaxing liquid (YA0031, Solarbio, China) for dewaxing. Following hydration with ethanol solutions, sections were heat-treated in sodium citrate antigen retrieval solution (Solarbio) for antigen retrieval. Subsequently, sections were fixed with 4% paraformaldehyde (G1101-500ML, Servicebio, China) for fixation, permeabilized with 0.3% Triton X-100 (IT9100, Solarbio, China), blocked with 10% normal goat serum (SL038, Solarbio, China), and incubated overnight at 4 °C with the primary antibody. The primary antibody required is consistent with Western Blot, with the addition of CD8 (1:100, Proteintech, 29896-1-AP, China) and Interferon gamma (1:50, Affinity, DF6045, China). The secondary antibody was incubated at room temperature for 1 hour. Nuclei were stained with DAPI for 10 minutes at room temperature, and samples were mounted with antifade medium.

The dewaxing, rehydration, and antigen retrieval procedures for immunohistochemical analysis were consistent with those for immunofluorescence analysis. After incubation with H_2_O_2_ for inactivation, samples were blocked with 5% BSA. Primary antibodies for Ki67 (Proteintech, 27309-1-AP, China) and Interferon gamma were incubated at room temperature for 1 hour, followed by secondary antibody incubation at room temperature for 30 minutes.

### 2.11 Flow cytometry analysis

After digesting animal tumor tissue with tissue digestion solution and filtering it, prepare a single-cell suspension. Incubate with the corresponding antibody at 4 °C in the dark for 30 minutes. Detect stained cells using a flow cytometer (CytoFLEX, BECKMAN, USA) and analyze the results with FlowJo software. Antibodies used included CD3 (100205, Biolegend, USA), CD4 (100405, Biolegend, USA), CD8a (100711, Biolegend, USA), IFN-γ (505815, Biolegend, USA), and Granzyme B Recombinant (396413, Biolegend, USA).

### 2.12 Statistical analysis

GraphPad Prism v10.0 software (La Jolla, CA, USA) and SPSS v26.0 software (IBM, Chicago, IL, USA) were used for statistical analysis. All *in vitro* experiments were performed independently at least three times. Comparisons between two groups were analyzed by a Student’s t-test. A one-way analysis of variance (ANOVA) with Bonferroni correction was used for comparisons between multiple groups. P < 0.05 was considered as statistical significance.

## 3 Result

### 3.1 Allicin inhibits the proliferation, invasion, and migration of osteosarcoma cells and reduces PD-L1 expression in these cells

**Figure 1:**
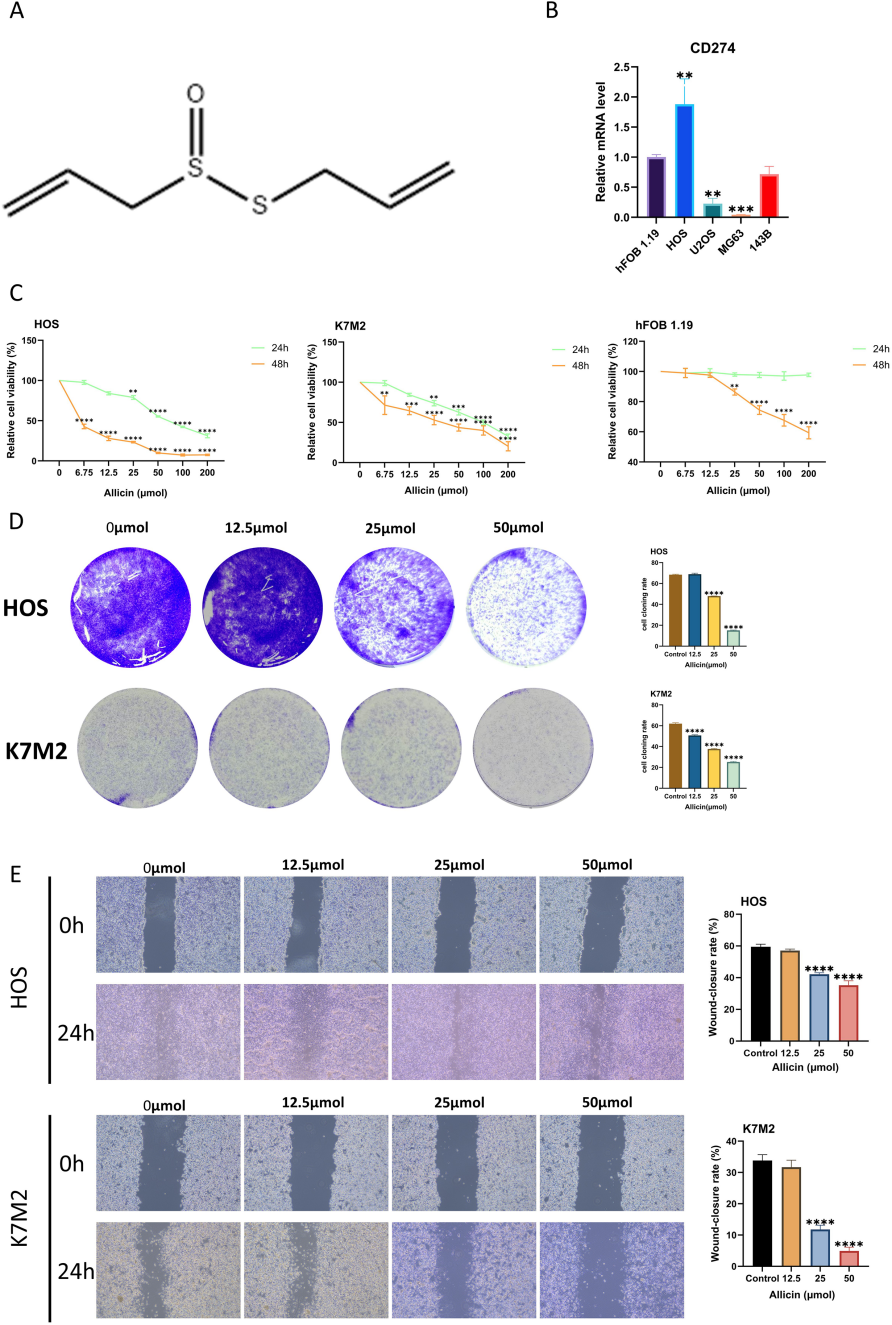
Allicin inhibits the growth and proliferation of osteosarcoma cells. The chemical structure of allicin is shown in Figure **(A)** The comparison of CD274 expression levels between the osteoblast cell line and various osteosarcoma cell lines was detected by RT-PCR **(B)**. The CCK-8 assay was employed to assess the effect of allicin on the proliferation of osteosarcoma cells and osteoblast cell line hFOB 1.19. The group treated with 0 μmol of allicin served as the control group. The asterisk (*) indicates statistically significant differences between each group and the control group **(C)**. The inhibitory effects of allicin on the growth and proliferation of osteosarcoma cells were validated through colony formation assays and scratch wound assays **(D, E)**. Results are expressed as mean ± SD (**p < 0.01, ***p < 0.001, ****p < 0.0001 vs. the control group).

**Figure 2:**
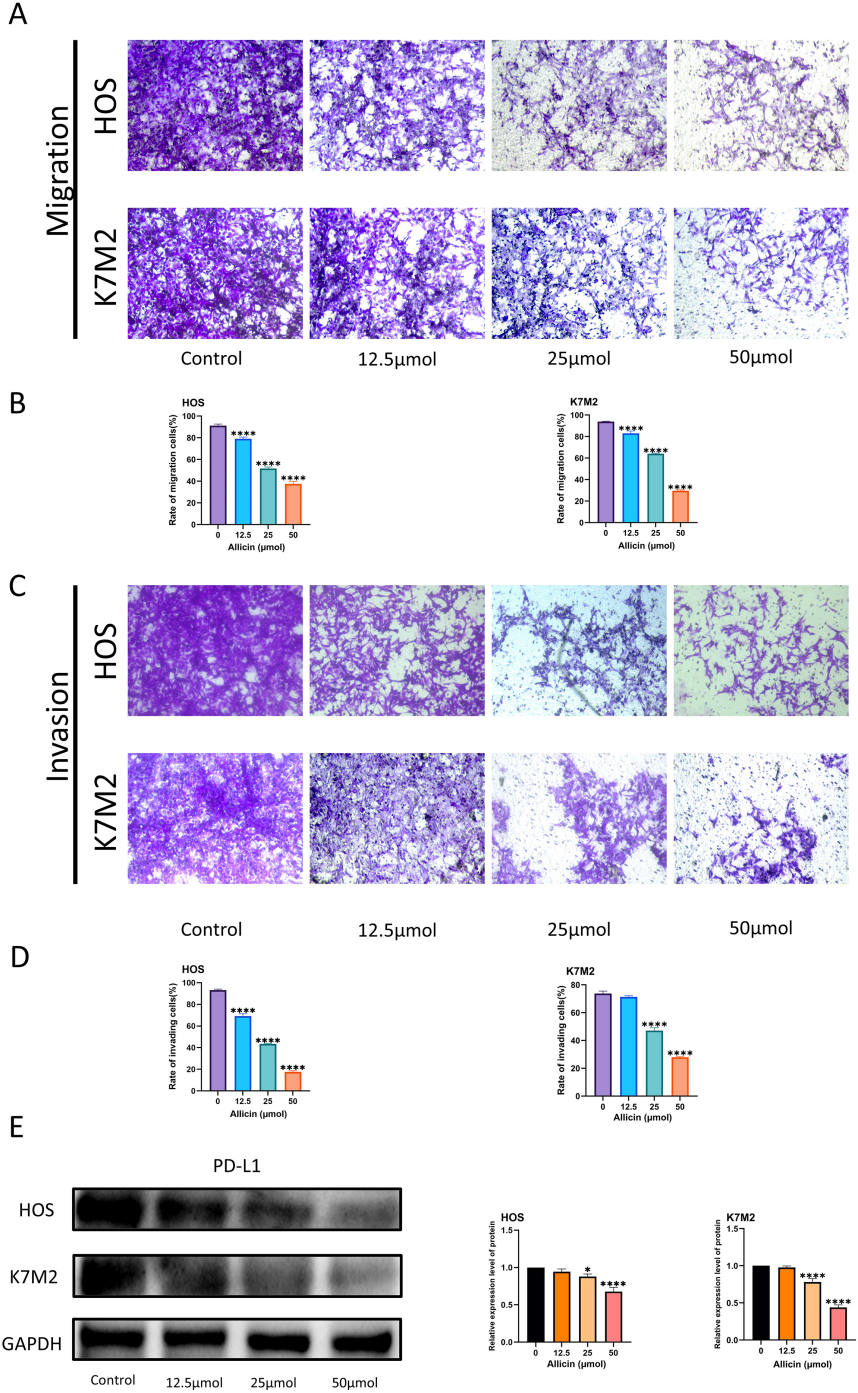
Allicin inhibits the migration and invasion of osteosarcoma cells and suppresses PD-L1 expression in these cells. Transwell assays were conducted to evaluate allicin’s inhibitory effects on osteosarcoma cell migration and invasion **(A–D)**. Western blot analysis was performed to detect PD-L1 protein expression in osteosarcoma cell lines following allicin treatment **(E)**. Results are expressed as mean ± SD (*p < 0.05, ****p < 0.0001 vs. the control group).

The structure of allicin is shown in **Figure 1A**. To select osteosarcoma cell lines suitable for this study, we assessed PD-L1 expression levels in untreated HOS, 143B, MG63, and U2OS cell lines. The human osteoblast cell line hFOB 1.19 was used as a control (**Figure 1B**). We found that PD-L1 expression was highest in the HOS cell line. This result is broadly consistent with the findings of Shen et al (17). Notably, osteoblasts exhibit higher PD-L1 expression than most osteosarcoma cell lines, a difference likely determined by the physiological characteristics of osteogenic differentiation. Having previously demonstrated allicin’s dose- and time-dependent inhibition of osteosarcoma cell viability, we further analyzed K7M2 and hFOB 1.19 cell lines using the CCK-8 assay. Optimal concentrations and durations were determined, with no adverse effect observed on normal osteoblasts (**Figure 1C**). In a clonogenic assay, allicin treatment significantly suppressed the colony-forming capacity of osteosarcoma cells (**Figure 1D**). The wound healing assay demonstrated that allicin significantly reduced the wound-healing ability of osteosarcoma cells in a dose-dependent manner (**Figure 1E**). Transwell assays demonstrated that allicin inhibited the migration and invasion of osteosarcoma cells in a dose-dependent manner (**Figures 2A–D**). We simultaneously measured PD-L1 expression levels in two osteosarcoma cell lines and found that allicin significantly inhibited PD-L1 expression (**Figure 2E**).

### 3.2 Investigating the mechanism of allicin’s inhibition of PD-L1

**Figure 3:**
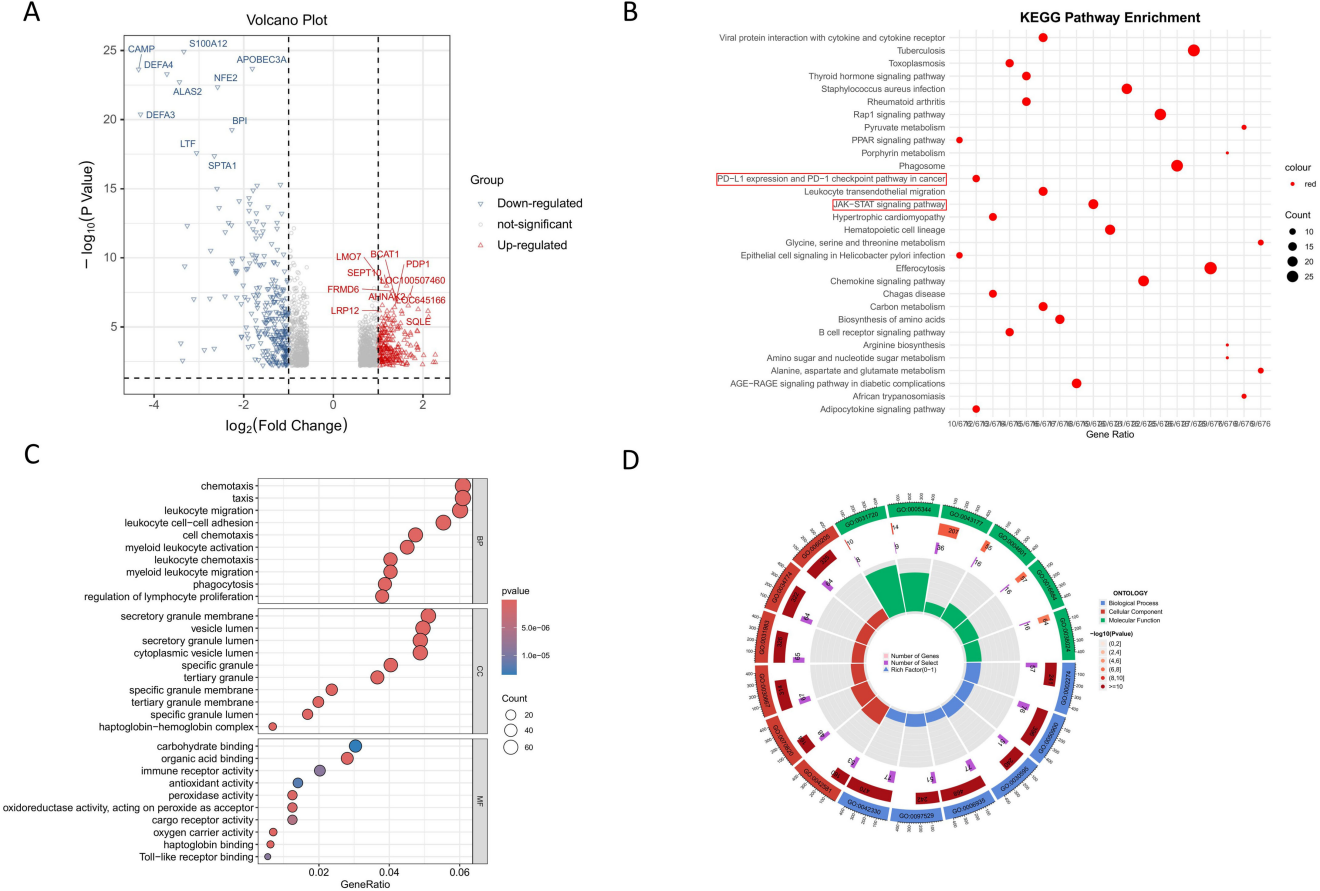
Mechanism of allicin-mediated PD-L1 inhibition in osteosarcoma cells based on bioinformatics analysis. The volcano plot shows differentially expressed genes associated with favorable prognosis in osteosarcoma patients **(A)**. Pathways significantly correlated with favorable prognosis identified through KEGG and GO pathway analysis **(B–D)**. A difference multiple of 1.5 or 2/3 was considered as different, and p < 0.05 was considered statistically significant.

We demonstrated the inhibitory effect of allicin on PD-L1 expression. To elucidate the mechanism underlying this effect, we conducted bioinformatics analysis. The volcano plot displayed differentially expressed genes associated with favorable prognosis in the GEO database chip GSE19276 (**Figure 3A**). KEGG and GO pathway analyses identified several pathways correlated with favorable prognosis, including PD-L1 expression and PD-1 checkpoint pathway in cancer, as well as the JAK-STAT signaling pathway (**Figures 3B–D**). Combining our findings with previous studies, we hypothesize that allicin suppresses PD-L1 expression in osteosarcoma cells via the IL-6/JAK2/STAT3 pathway (18).

### 3.3 Allicin inhibits the IL-6/JAK2/STAT3 axis in osteosarcoma cells and ultimately suppresses PD-L1 expression

**Figure 4:**
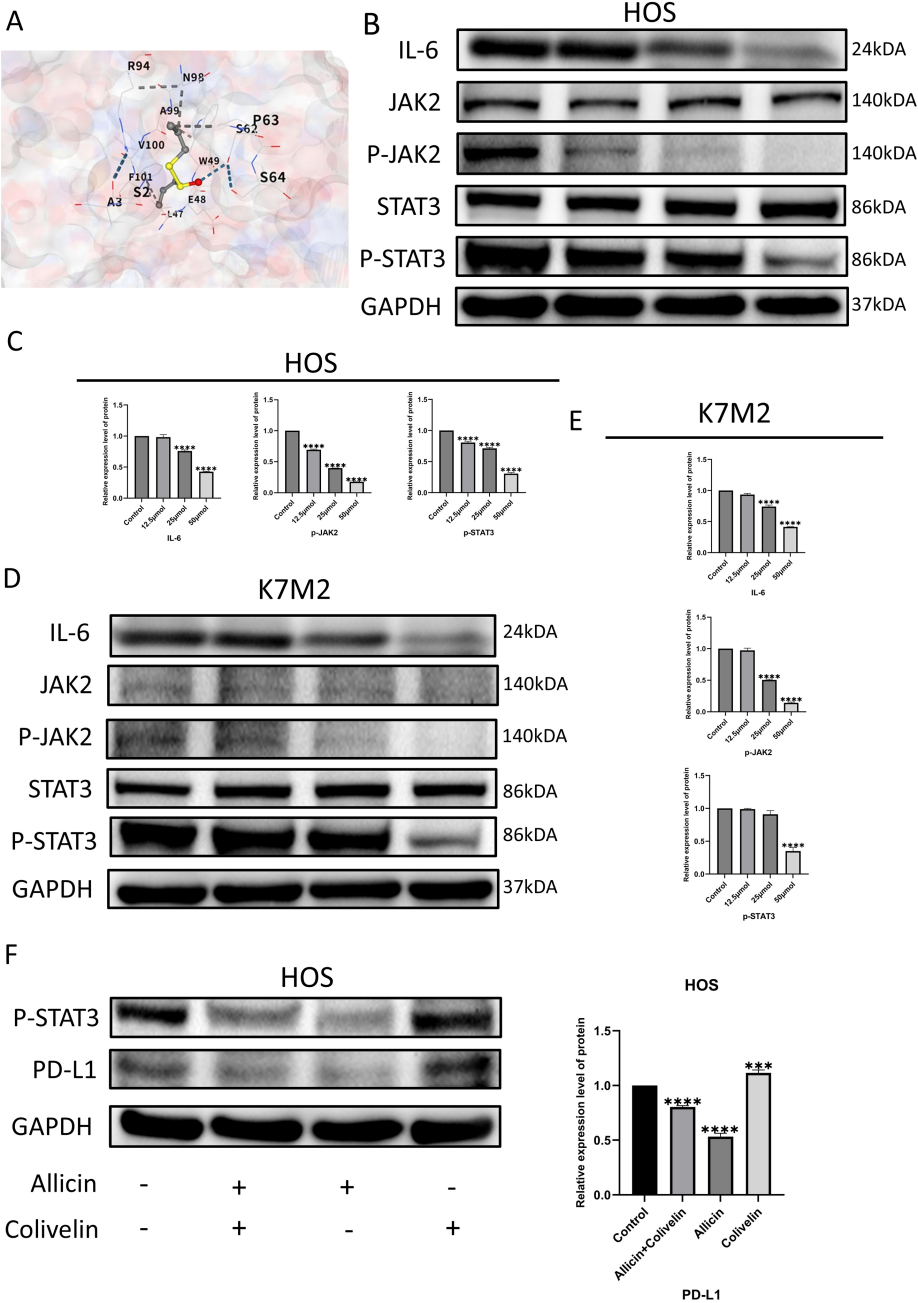
Allicin inhibits the IL-6/JAK2/STAT3 axis in osteosarcoma cells and ultimately suppresses PD-L1 expression. Molecular Docking of Allicin and IL-6 **(A)**. Western blot analysis of IL-6/JAK2/STAT3 axis protein expression in HOS and K7M2 cell lines **(B–E)**. Expression levels of phosphorylated STAT3 and PDL1 proteins in HOS cells following Colivelin (phosphorylated STAT3 agonist) treatment **(F)**. Results are expressed as mean ± SD (***p < 0.001, ****p < 0.0001 vs. the control group).

To further validate our hypothesis, we employed molecular docking technology and discovered that allicin exhibits strong binding affinity to the IL-6 protein (**Figure 4A**). We employed Western blot analysis to assess the expression levels of pathway proteins including IL-6, phosphorylated JAK2, and phosphorylated STAT3 (**Figures 4B–E**). In the allicin-treated group, the expression levels of phosphorylated JAK2 and phosphorylated STAT3 in osteosarcoma cells were significantly suppressed. This demonstrates that allicin indeed inhibits the expression of the IL-6/JAK2/STAT3 pathway. To further determine whether allicin modulates PD-L1 by inhibiting this pathway, we treated both allicin-treated and untreated HOS osteosarcoma cell lines with Colivelin. Western blot analysis revealed that Colivelin-treated cells exhibited significantly elevated phosphorylated STAT3 expression compared to untreated cells. Consequently, PD-L1 protein transcription increased, leading to heightened expression. Concurrently, in cells treated with allicin, Colivelin reversed the inhibition of phosphorylated STAT3 induced by allicin, partially restoring PD-L1 expression (**Figure 4F**). Integrating these findings, we conclude that allicin suppresses PD-L1 expression by inhibiting the IL-6/JAK2/STAT3 pathway.

### 3.4 Allicin inhibits immune evasion in mouse osteosarcoma and improves the tumor microenvironment

**Figure 5:**
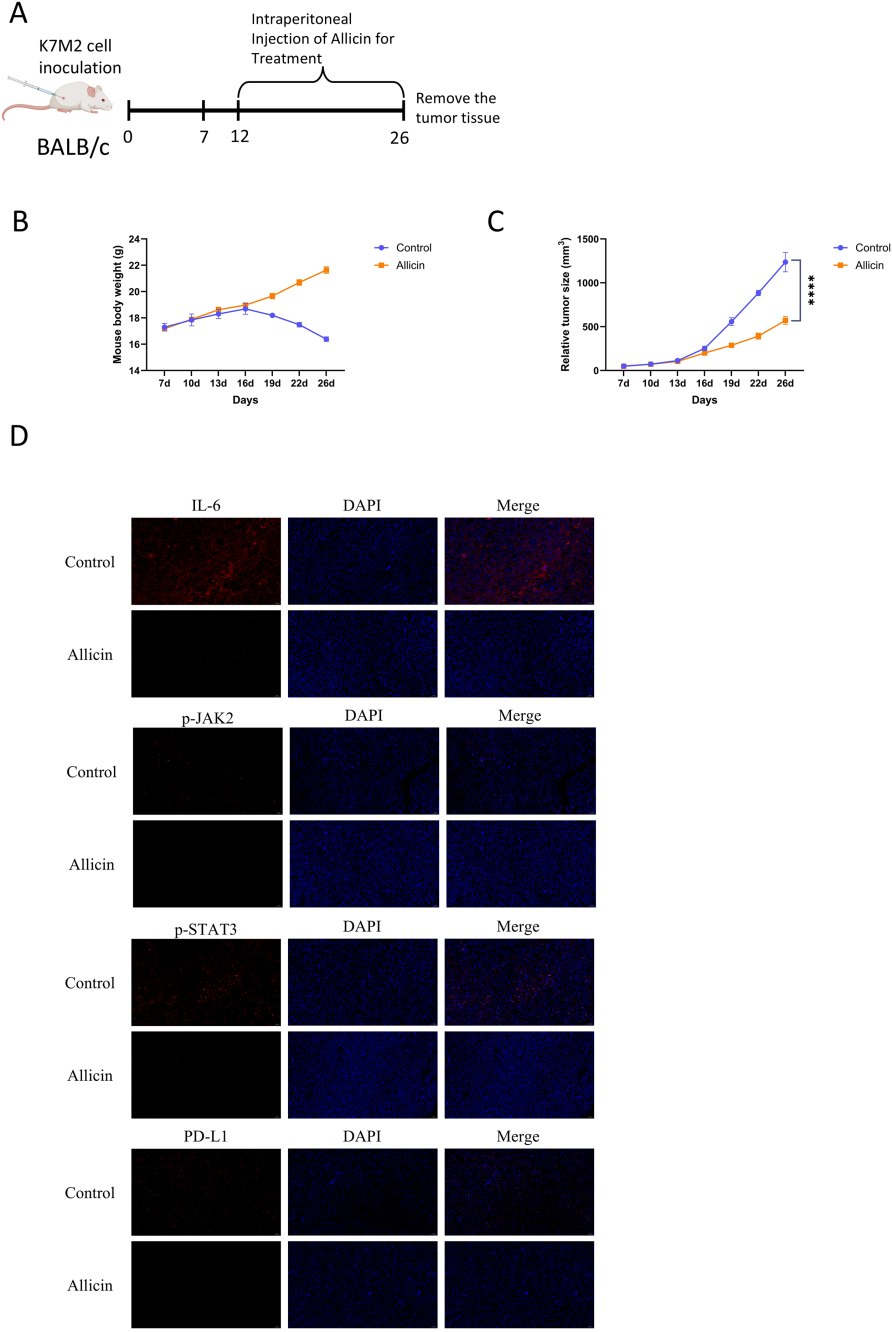
Allicin inhibits tumor proliferation in mice and suppresses the IL-6/JAK2/STAT3 pathway. Tumors were implanted in BALB/c mice using K7M2 cells. After 14 days of Allicin intervention, tissue samples were collected **(A)**. Body weights and tumor volumes were compared between the allicin-treated group and the control group **(B, C)**. Immunofluorescence staining was performed to detect the expression of IL-6, p-JAK2, p-STAT3, and PD-L1 in tumor sections **(D)**. Scale bar = 50 μm. Results are expressed as mean ± SD (****p < 0.0001 vs. the control group).

**Figure 6:**
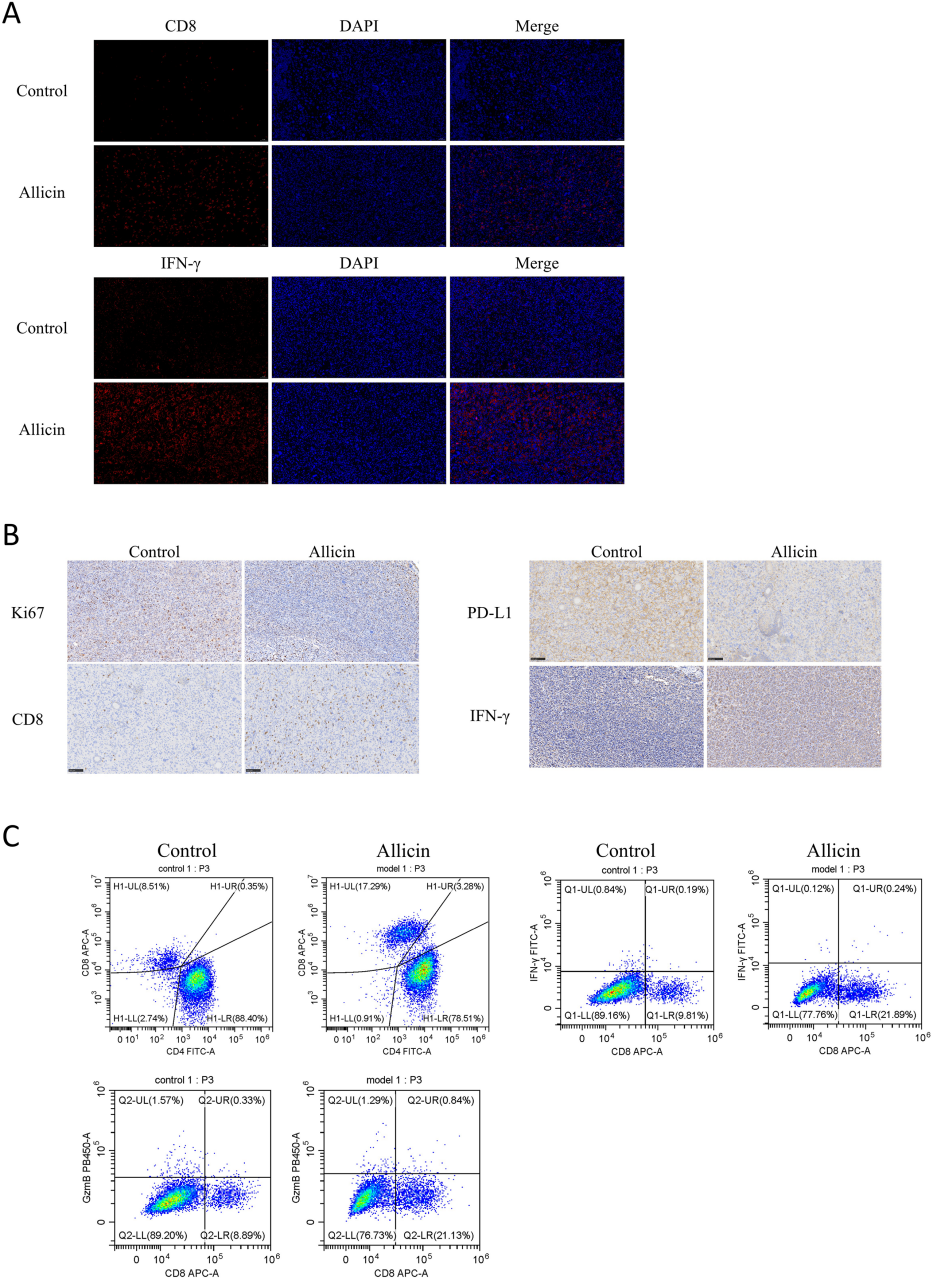
Allicin treatment enhances the immune response to tumors and improves the tumor microenvironment. Immunofluorescence assay for detecting CD8 and IFN-γ expression. **(A)** Immunohistochemical staining for Ki67, CD8, PD-L1, and IFN-γ expression. **(B)** Flow cytometry for CD3+, CD4+, and CD8+ T cell counts and proportions, IFN-γ, and granzyme **(C)**. Scale bar = 50 μm.

We have demonstrated that allicin suppresses the expression of the immune checkpoint PD-L1. To further validate allicin’s ability to inhibit immune evasion in osteosarcoma, we established an osteosarcoma model in BALB/c mice. The mice were divided into two groups: a control group and an allicin-treated group. Analysis of mouse body weight and tumor volume revealed that allicin exhibited favorable therapeutic effects against osteosarcoma (**Figures 5A–C**). We first employed immunofluorescence to detect the expression of the IL-6/JAK2/STAT3 pathway and PD-L1 protein (**Figure 5D**). Results demonstrated that allicin effectively suppressed activation of the JAK/STAT pathway, thereby inhibiting PD-L1 expression. To further validate suppression of immune evasion following PD-L1 blockade, immunofluorescence was used to detect CD8+ T cells and IFN-γ (**Figure 6A**). Immunohistochemical analysis confirmed this conclusion: the proliferative capacity of mouse tumors decreased, while their immune response capability significantly increased (**Figure 6B**). Following the aforementioned examination, allicin treatment resulted in a significant increase in CD8+ expression within tumor tissues. To further examine the immune microenvironment, flow cytometry analysis of tumor T lymphocyte subsets and immune function expression markers revealed that allicin treatment effectively promoted the recruitment of both CD4+ and CD8+ T cells while enhancing the expression of IFN-γ and granzyme B (**Figure 6C**). This indicates that allicin indeed promotes tumor immune responses, improves the tumor microenvironment, and enhances the cytotoxic activity of immune cells.

## 4 Discussion

Survival rates for patients with metastatic or recurrent osteosarcoma remain low, largely due to the development of resistance to conventional chemotherapy. Consequently, the development of new treatment approaches has become a consensus (19). Since the development of immunotherapy, it has been applied to numerous tumors and achieved remarkable clinical outcomes, effectively extending survival in patients with melanoma, non-small cell lung cancer, and other cancers (20). Immunotherapy approaches, including CAR-modified cellular therapies and immune checkpoint inhibitors (ICIs) treatments represented by PD-L1 and CTLA-4 blockade, are now being researched for the treatment of osteosarcoma (21). Regrettably, although PD-1/PD-L1 axis blockade demonstrates antitumor activity in mouse models (22), most patients in clinical trials have not shown clinical benefit (23). This may be attributed to the unique suppressive tumor microenvironment characteristic of human osteosarcoma. Therefore, developing novel therapeutic agents that combine with ICIs to improve the tumor microenvironment and enhance immunotherapy efficacy may offer a solution to this challenge.

**Figure 7:**
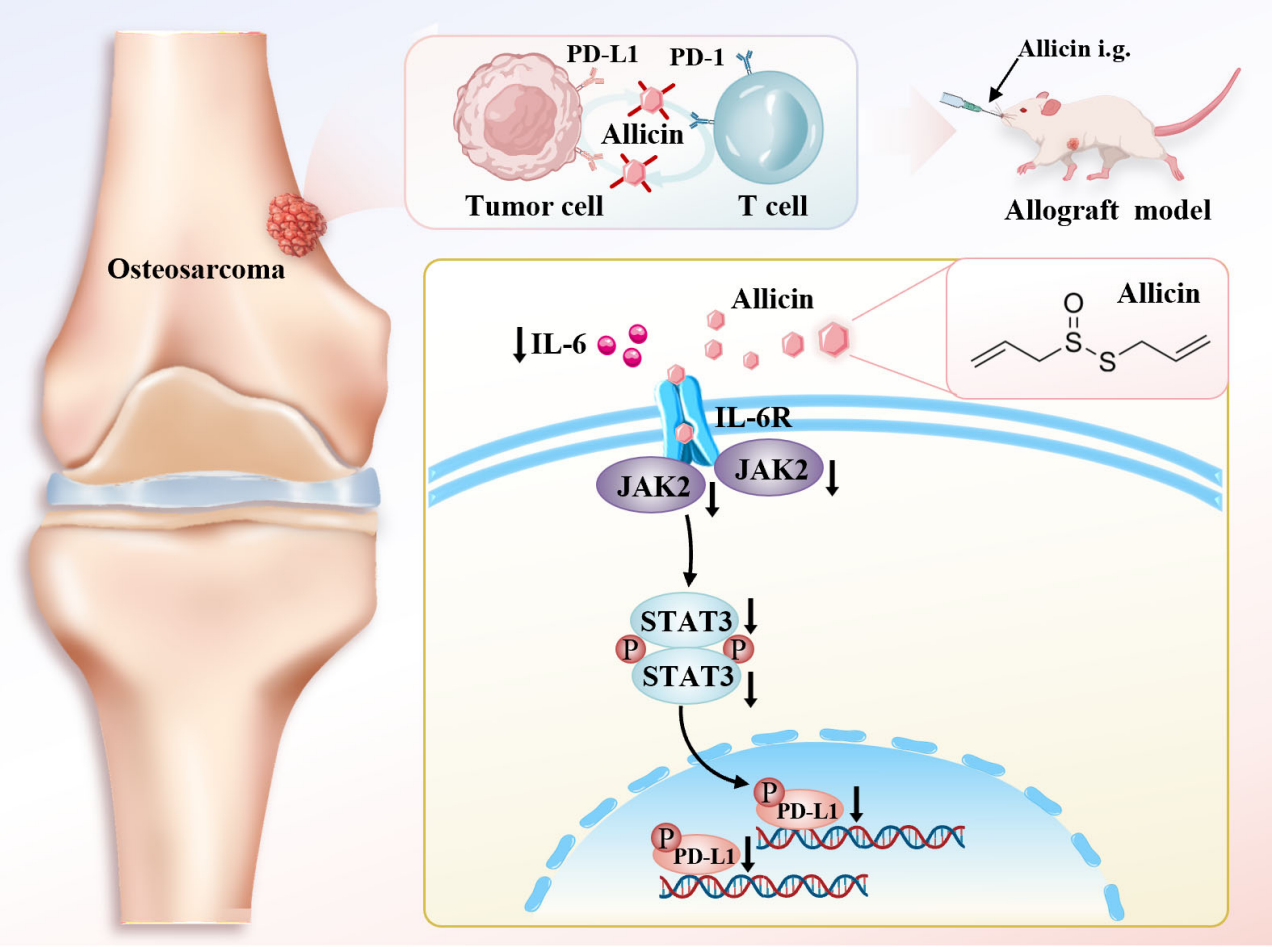
Allicin inhibits PD-L1 protein expression in osteosarcoma cells via the IL-6/JAK2/STAT3 pathway. It suppresses PD-1/PD-L1-mediated immune evasion in BALB/c mice and improves the tumor microenvironment.

Natural products have played a significant role in the history of anticancer drug development (24). Allicin has long been applied to combat infections caused by pathogens such as bacteria, fungi, and viruses. As a natural antibiotic, it plays a role in boosting immunity (25). Our team has demonstrated for the first time that allicin, a natural product, inhibits osteosarcoma immune evasion and modulates the immune microenvironment. This finding partially aligns with observations from a recent study by Jie et al (26). In this research, we aimed to validate how allicin modulates immunity in osteosarcoma and the mechanisms underlying this process (**Figure 7**).

Consistent with our previous studies, assays including colony formation, scratch wound, and Transwell assays demonstrated that allicin potently inhibits the growth, migration, and invasion of osteosarcoma cells, exhibiting potential as an antitumor drug. In a prior study, researchers discovered that allicin can reverse T-cell exhaustion, and in-silico design studies concluded that allicin may act as a PD-L1 inhibitor. However, subsequent experiments failed to validate these findings (27). Our research provides the first evidence through Western blot analysis that PD-L1 expression is inhibited in a concentration-dependent manner with allicin. Furthermore, we sought to elucidate the mechanism by which allicin regulates PD-L1. Our experimental results, combined with bioinformatics analysis, revealed that allicin inhibits the JAK2/STAT3 pathway. While the inhibitory effect of allicin on the JAK/STAT axis is not a novel discovery (28), we are the first to demonstrate that allicin can suppress PD-L1 through this pathway. JAK2 is an intracellular non-receptor tyrosine kinase belonging to the Janus kinase/signal transducer and activator of transcription (JAK/STAT) family. It participates in multiple biological processes including cell proliferation and apoptosis, immune function, and angiogenesis (29). JAK2 is abnormally activated in tumors, leading to continuous activation of STAT3. Phosphorylated STAT3 then promotes PD-L1 transcription (7, 30). In osteosarcoma treated with allicin, both the expression of phosphorylated JAK2 and phosphorylated STAT3 were correspondingly reduced.

Research on the interaction between IL-6 and PD-L1 has been increasing year by year, revealing a complex relationship between the two. However, it is certain that IL-6 enhances the functional expression of PD-L1 through multiple pathways, driving immune escape (31, 32). Among these pathways is the JAK2/STAT3 pathway (33). Combined blockade of IL-6 and PD-L1 has demonstrated superior antitumor activity (34). As demonstrated by the results, molecular docking technology revealed that allicin exhibits strong binding affinity to IL-6 and exerts significant inhibitory effects. Combined with Western blot analysis, allicin can inhibit IL-6 protein expression in osteosarcoma cells in a concentration-dependent manner. Based on this, we conclude that allicin’s pronounced regulatory effect on PD-L1 arises precisely through its inhibition of IL-6, which in turn suppresses the JAK2/STAT3 pathway.

A growing body of research indicates that PDL1, in addition to participating in tumor immune evasion mechanisms, possesses several other independent functions. It can promote epithelial-mesenchymal transition (EMT) programs through the PI3K-AKT and MAPK pathways (35), and support tumor progression via the mTOR pathway (36). This study indirectly confirms that allicin inhibits PD-L1, thereby exerting antitumor effects.

The significance of blocking immune escape lies in activating the immune response in cancer patients. The recruitment of cytokines and immune cells serves as a hallmark of this immune response activation (37). CD8+ T cells are the primary cells responsible for antitumor activity, while CD4+ T cells were initially thought to perform auxiliary functions such as activating the immune response and supporting antitumor effects (38). However, recent research supports the critical role of CD4+ cells in exerting sustained anti-tumor effects and improving the tumor microenvironment (39, 40). Our flow cytometry analysis revealed a significant increase in the recruitment of CD8+ and CD4+ T cells within tumors in mice treated with allicin. The CD8+/CD3+ and CD4+/CD3+ ratios also increased. These ratios are considered markers of enhanced immune function (41). Interferon-gamma (IFN-γ) is one of the key factors determining the efficacy of immunotherapy. Secreted by T lymphocytes, it plays a crucial role in activating the immune response (42). Additionally, IFN-γ possesses independent antitumor effects by inducing cell death through apoptosis and necroptosis, and it can promote tumor cell senescence (43). Granzyme B is one of the primary substances secreted by CD8+ T cells that exert antitumor effects (44). It is also utilized to assess the tumor immune microenvironment (45). Our flow cytometry analysis similarly revealed that allicin treatment increased both interferon-γ and granzyme B levels within mouse tumors. Based on these findings, our research indicates that allicin possesses the potential to inhibit immune evasion in mouse osteosarcoma and treat mouse osteosarcoma, while also showing promise in improving the immune microenvironment of mouse osteosarcoma.

## 5 Conclusion

In conclusion, allicin suppresses PD-L1 expression in osteosarcoma by inhibiting the IL-6/JAK2/STAT3 pathway, thereby counteracting immune evasion in this malignancy. Concurrently, we observed improvements in the associated immune microenvironment. These findings demonstrate allicin’s potential value as an adjunct to immunotherapy.

## Data Availability

The original contributions presented in the study are included in the article/**Supplementary Material**. Further inquiries can be directed to the corresponding authors.
