# Supplementary material for: Allicin inhibits PD-L1 through the IL-6/JAK2/STAT3 pathway to suppress immune evasion in osteosarcoma
Source: Front Immunol. 2026 Feb 20;17:1735090. doi: 10.3389/fimmu.2026.1735090 (PMC12962910; doi:10.3389/fimmu.2026.1735090)
Supplement: Supplementary file 1 [file DataSheet1.pdf]

HOS-PDL1

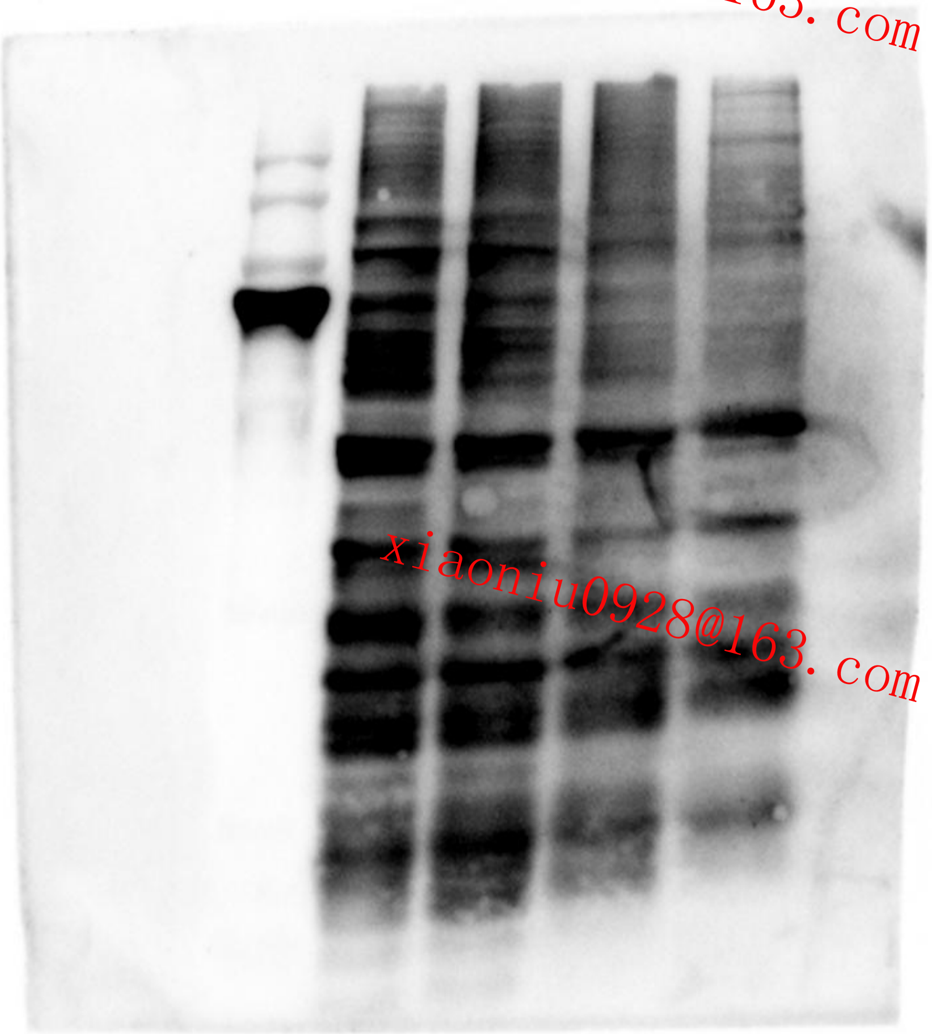

xiaoniu0928@163.com

HOS-PDL1-GAPDH

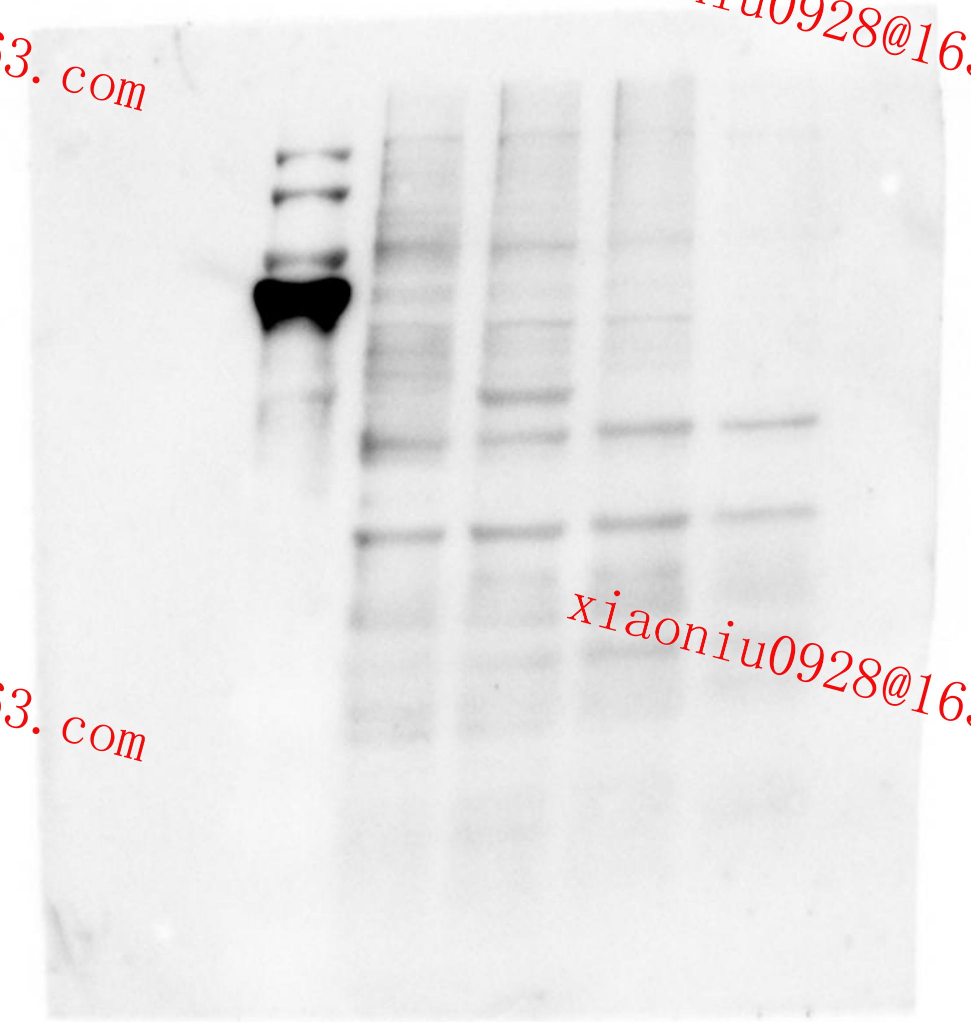

xiaoniu0928@163.com
xiaoniu0928@163.com
xiaoniu0928@163.com
xiaoniu0928@163.com
xiaoniu0928@163.com
xiaoniu0928@163.com

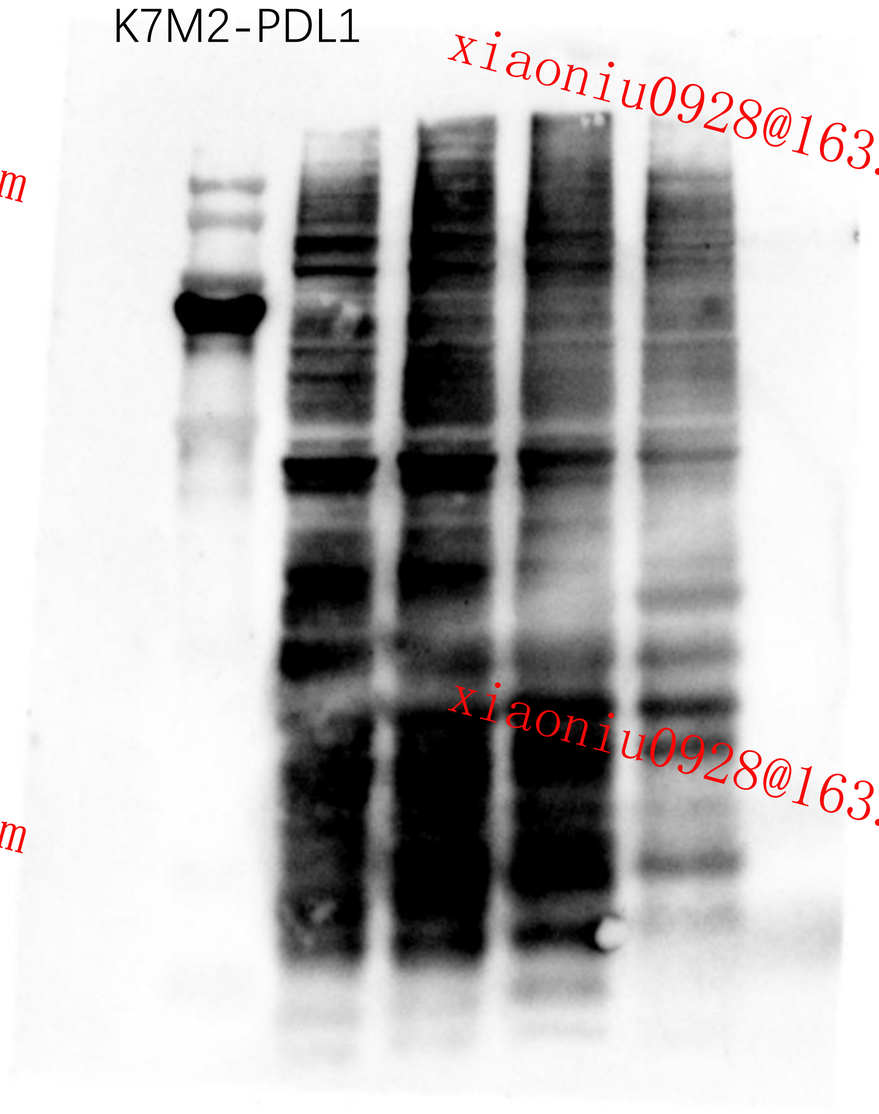

xiaoniu0928@163.com
xiaoniu0928@163.com
xiaoniu0928@163.com
xiaoniu0928@163.com
xiaoniu0928@163.com
xiaoniu0928@163.com

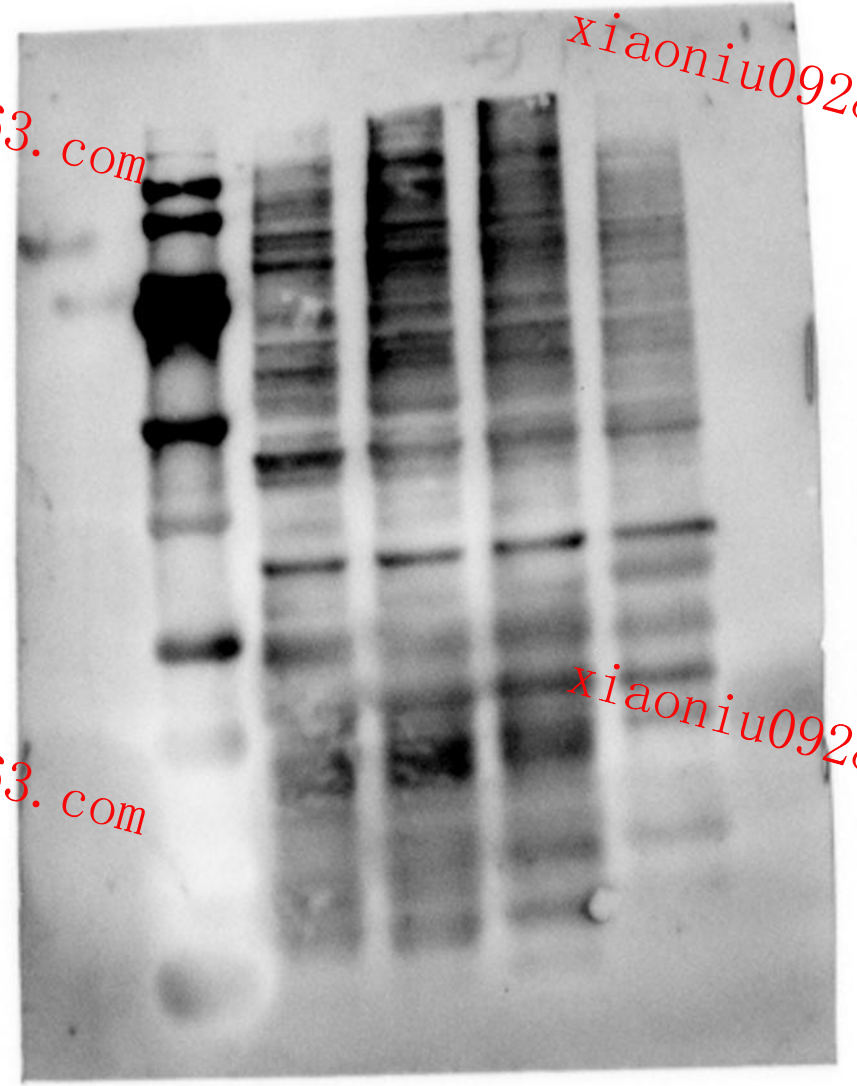

K7M2-PDL1-GAPDH

xiaoniu0928@163.com

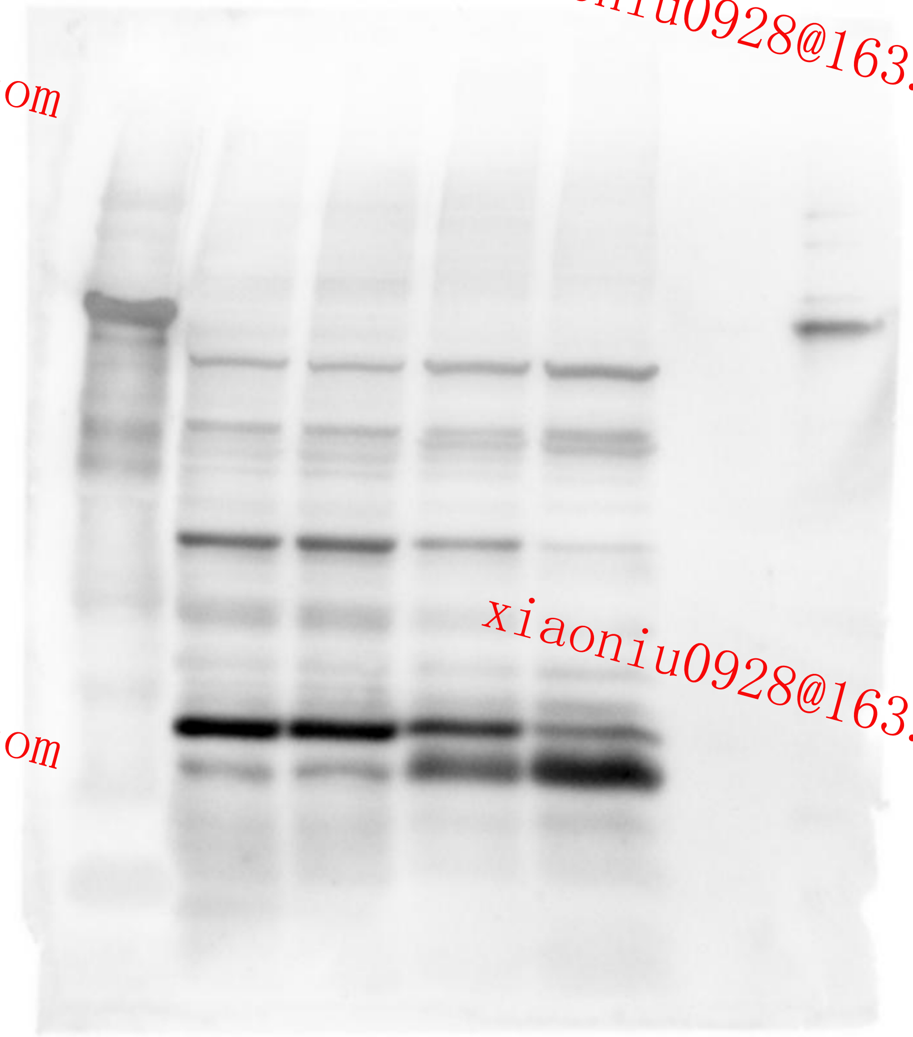

xiaoniu0928@163.com

HOS-IL6-GAPDH

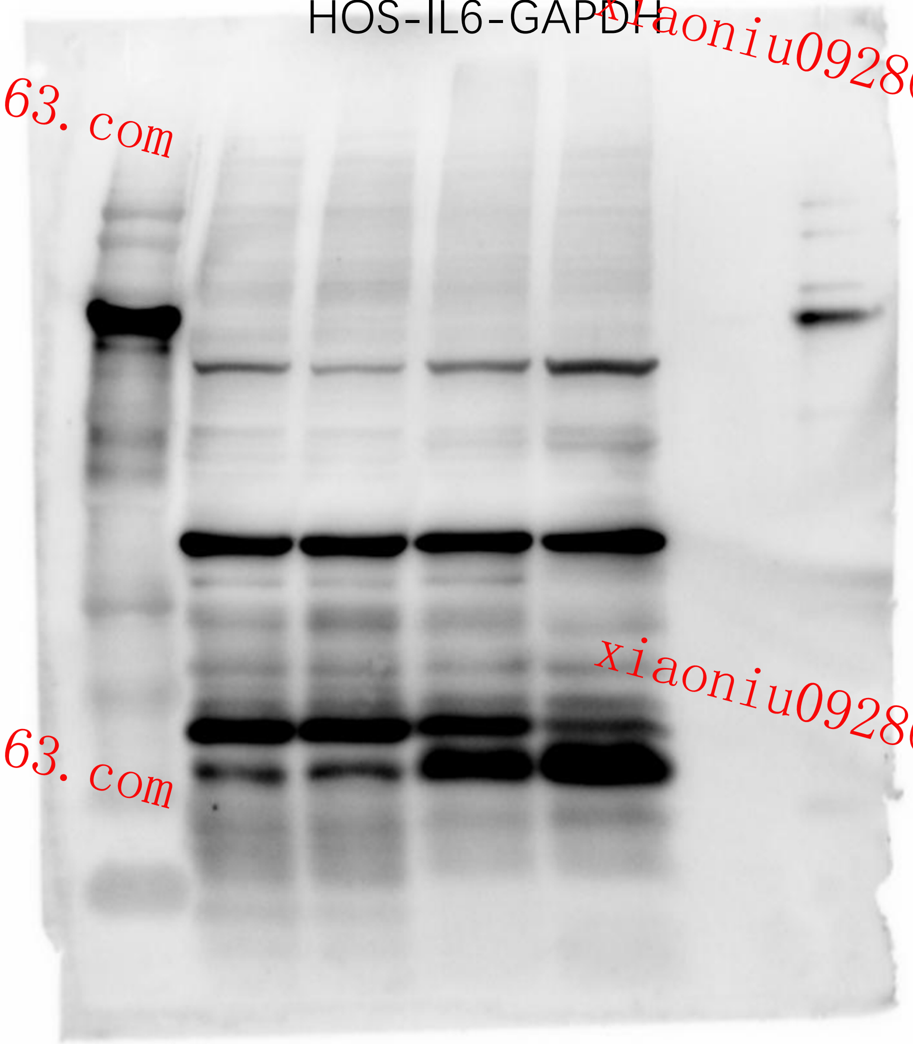

xiaoniu0928@163.com

K7M2-IL6

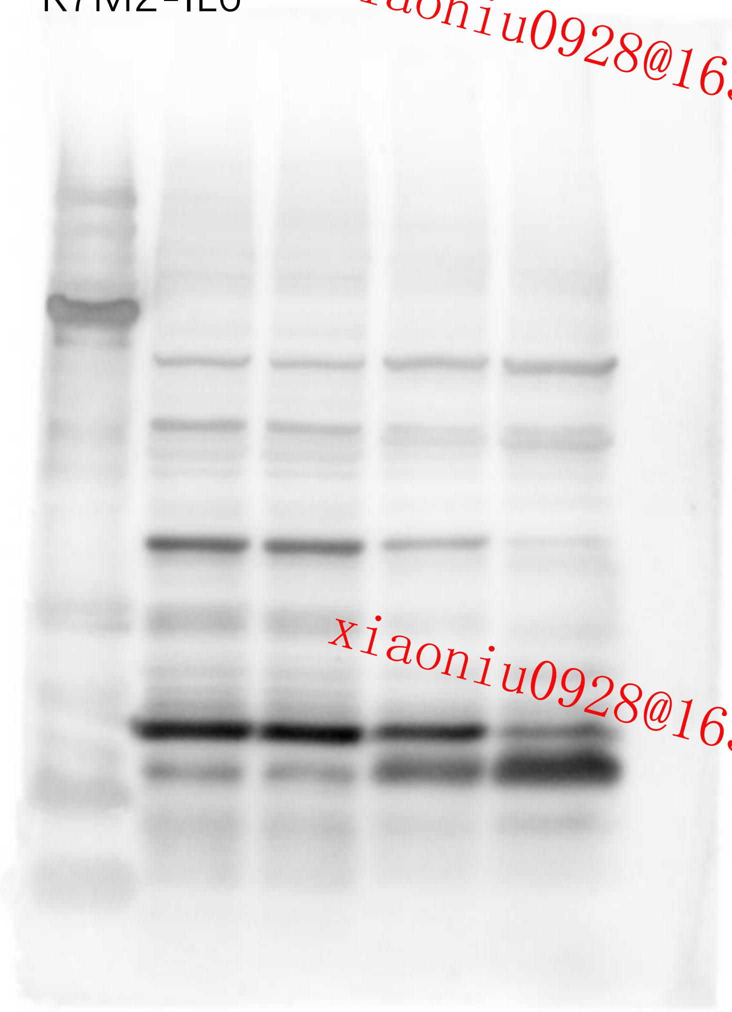

xiaoniu0928@163.com

K7M2-IL6-GAPDH

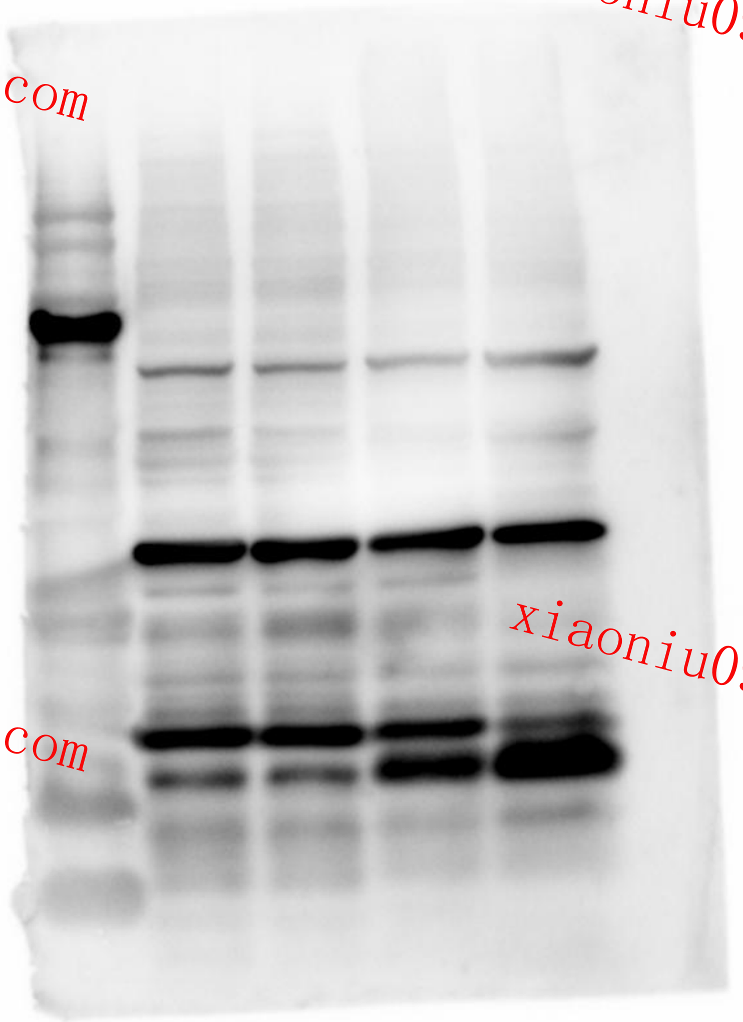

xiaoniu0928@163.com
xiaoniu0928@163.com
xiaoniu0928@163.com
xiaoniu0928@163.com
xiaoniu0928@163.com
xiaoniu0928@163.com

HOS-JAK2

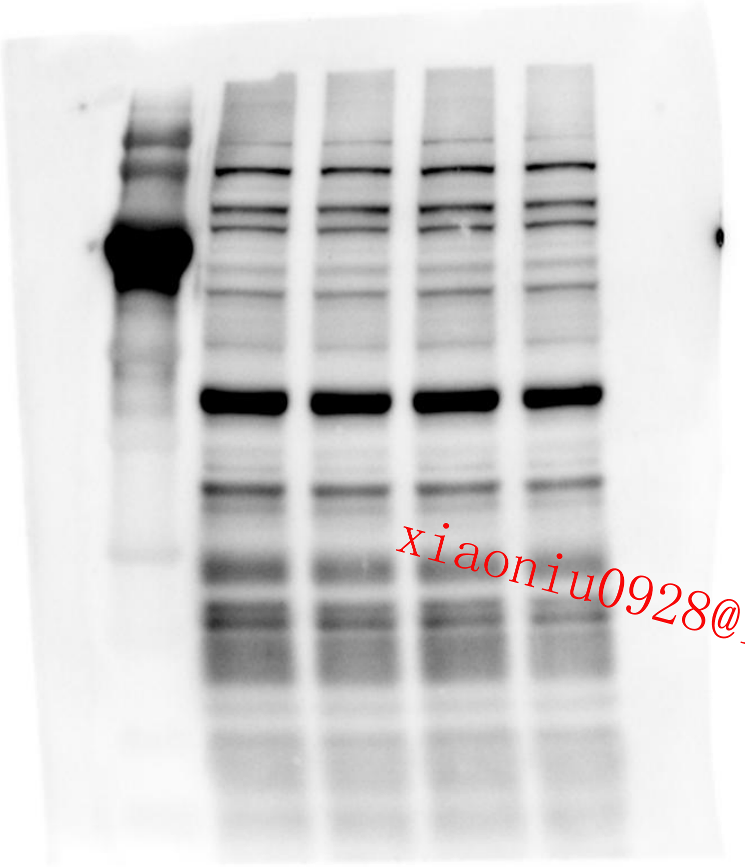

xiaoniu0928@163.com
xiaoniu0928@163.com
xiaoniu0928@163.com
xiaoniu0928@163.com
xiaoniu0928@163.com
xiaoniu0928@163.com

HOS-JAK2-GAPDH

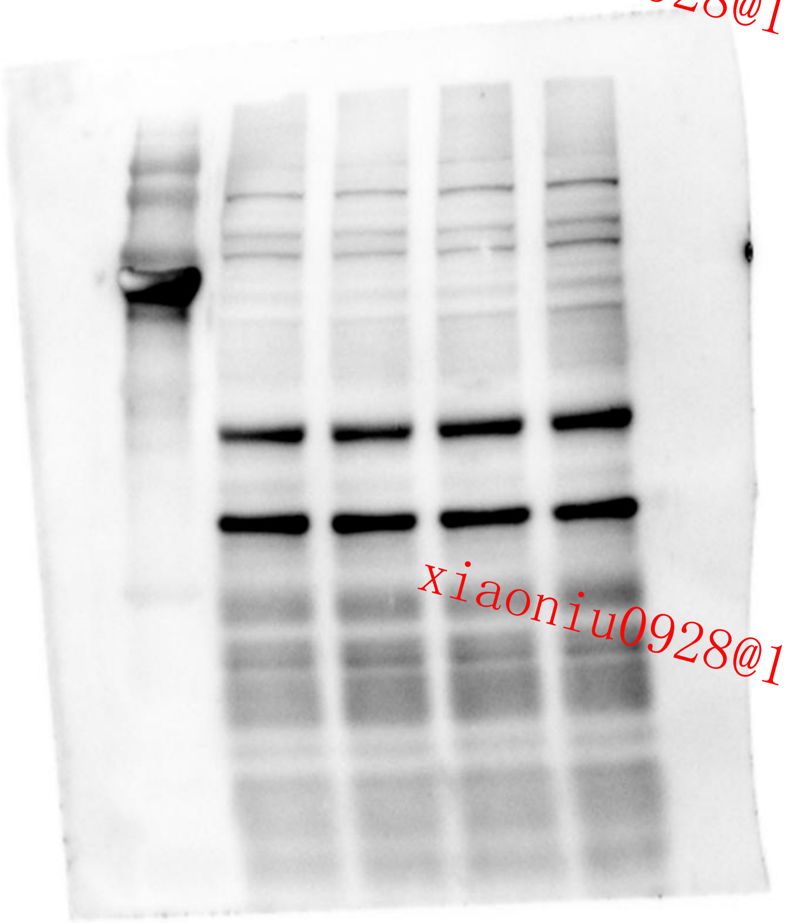

xiaoniu0928@163.com xiaoniu0928@163.com xiaoniu0928@163.com
xiaoniu0928@163.com xiaoniu0928@163.com xiaoniu0928@163.com

K7M2-JAK2

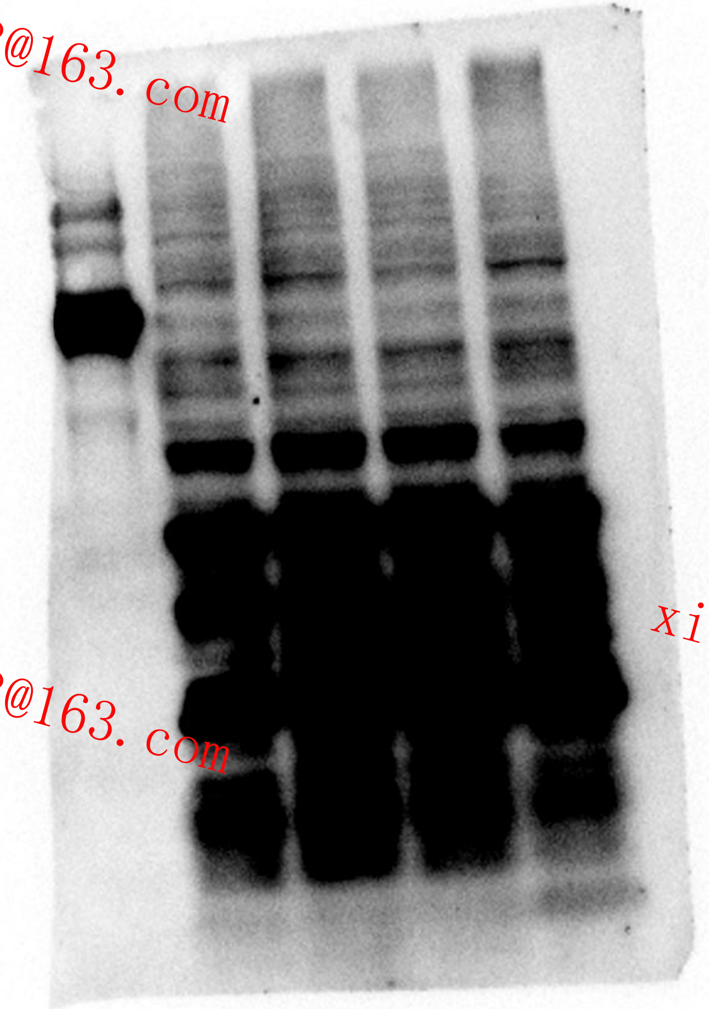

xiaoniu0928@163.com xiaoniu0928@163.com xiaoniu0928@163.com
xiaoniu0928@163.com xiaoniu0928@163.com xiaoniu0928@163.com

K7M2-JAK2-GAPDH

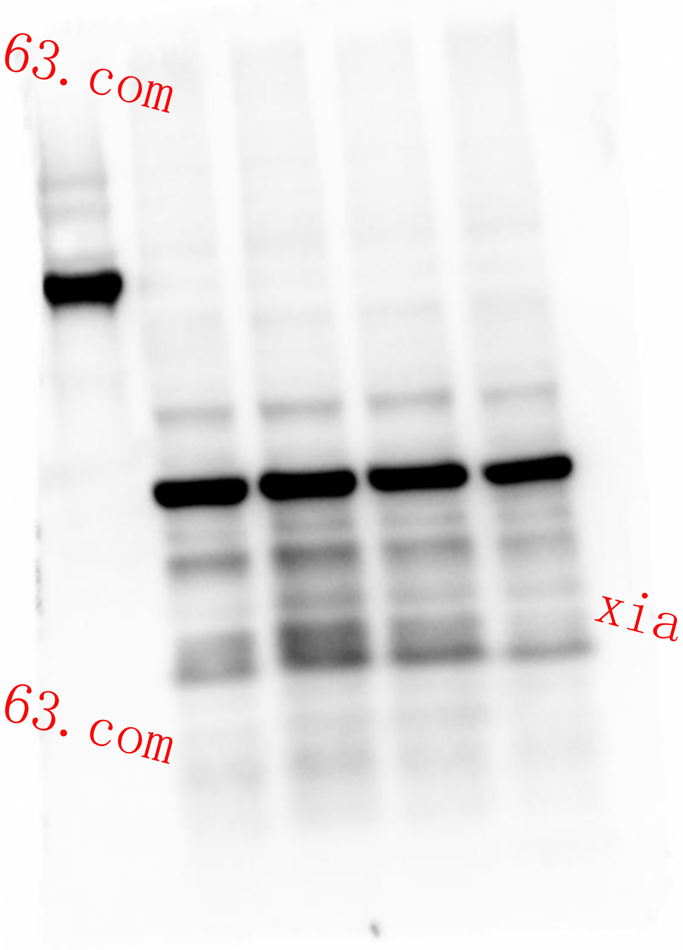

xiaoniu0928@163.com
xiaoniu0928@163.com
xiaoniu0928@163.com
xiaoniu0928@163.com
xiaoniu0928@163.com
xiaoniu0928@163.com

HOS-pJAK2

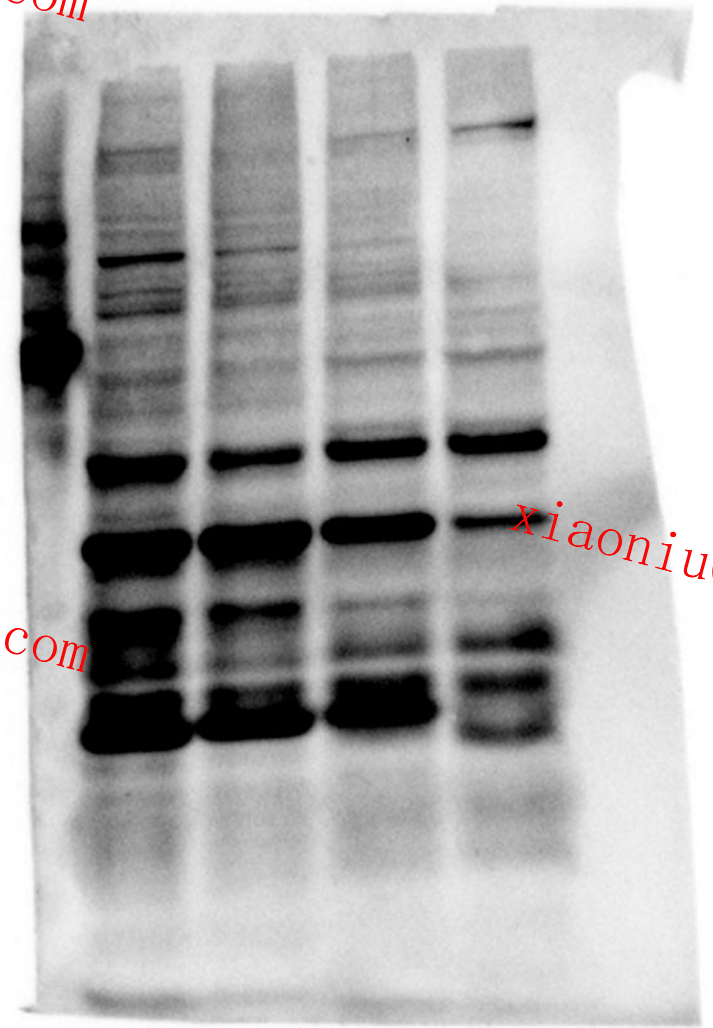

xiaoniu0928@163.com

HOS-pJAK2-GAPDH

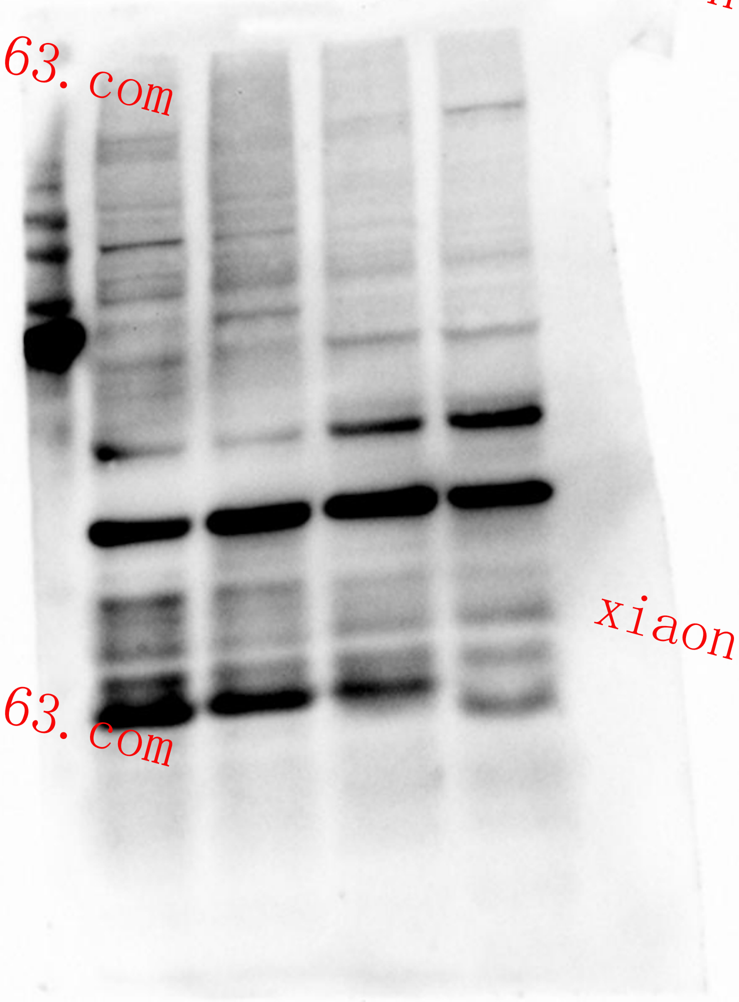

xiaoniu0928@163.com xiaoniu0928@163.com xiaoniu0928@163.com
xiaoniu0928@163.com xiaoniu0928@163.com xiaoniu0928@163.com

K7M2-pJAK2

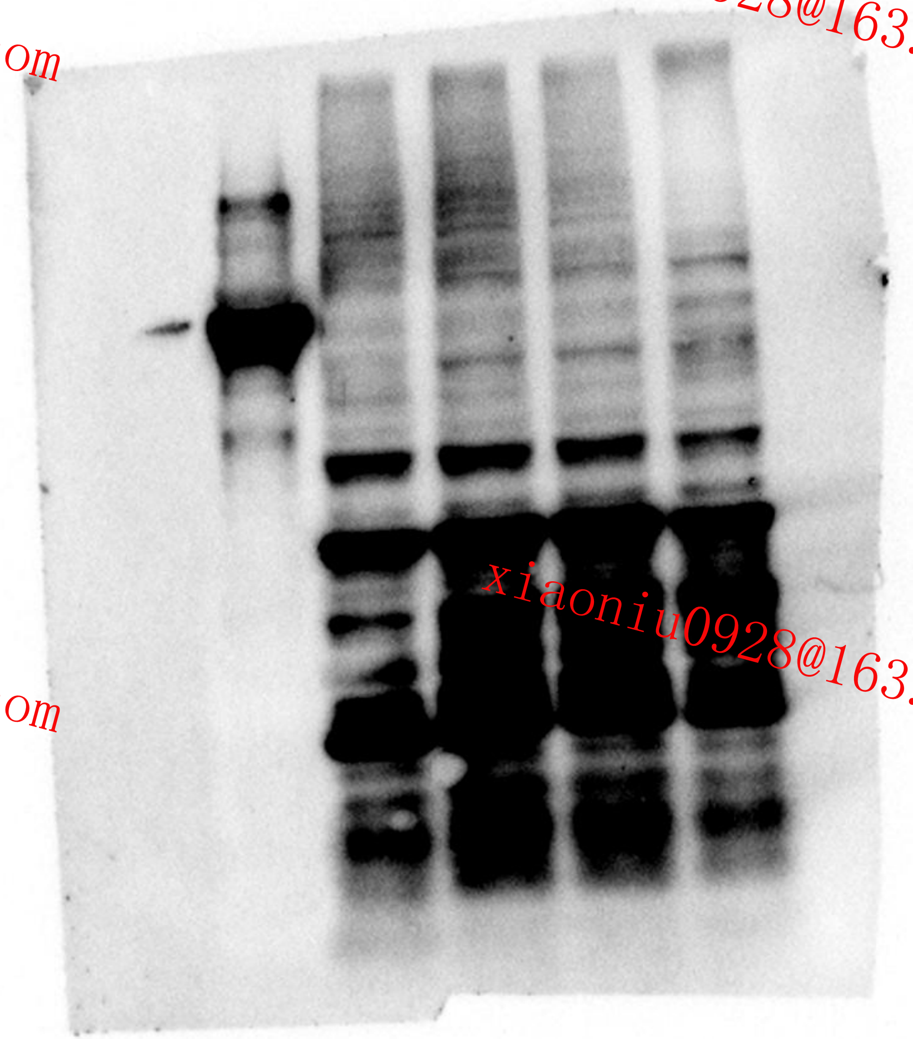

xiaoniu0928@163.com

K7M2-pJAK2-GAPDH

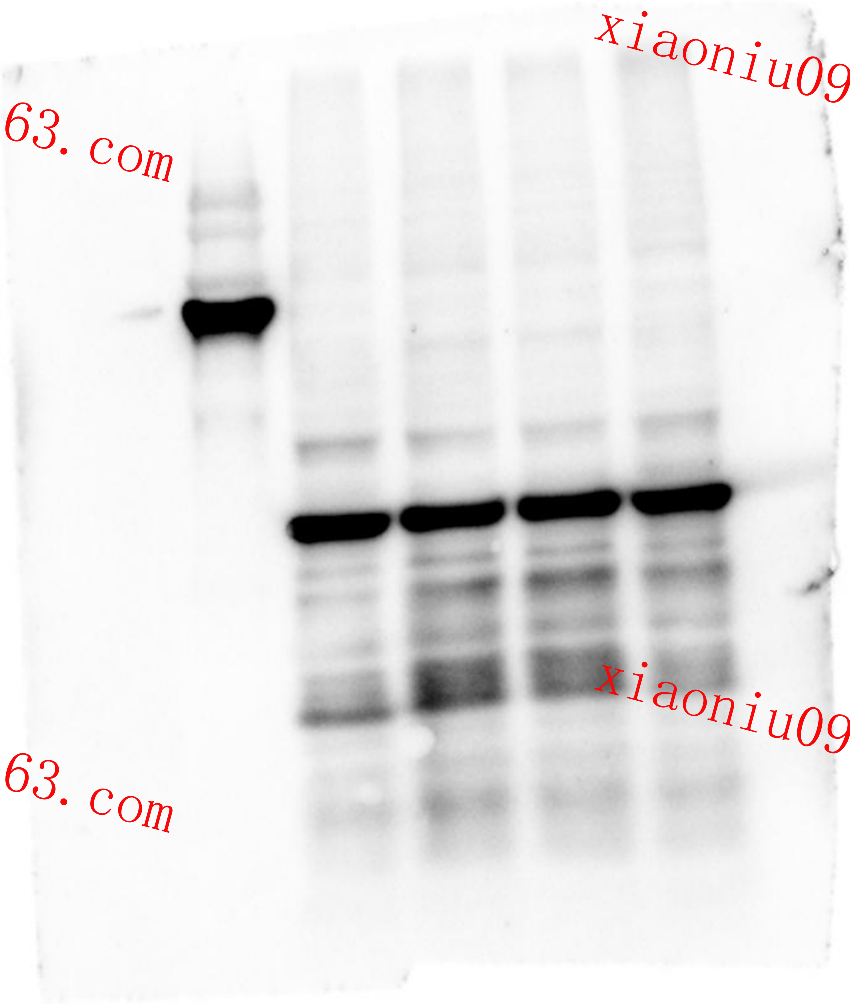

xiaoniu0928@163.com xiaoniu0928@163.com xiaoniu0928@163.com
xiaoniu0928@163.com xiaoniu0928@163.com xiaoniu0928@163.com

HOS-STAT3

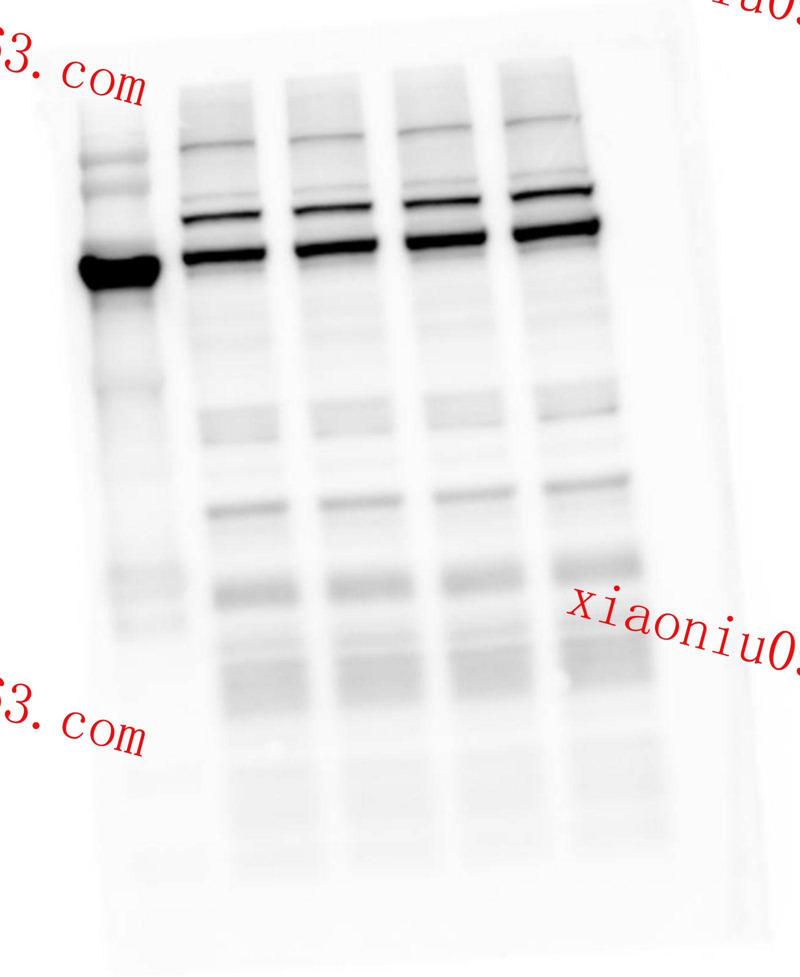

xiaoniu0928@163.com xiaoniu0928@163.com xiaoniu0928@163.com
xiaoniu0928@163.com xiaoniu0928@163.com xiaoniu0928@163.com
xiaoniu09[illegible] xiaoniu09[illegible] xiaoniu09[illegible]

HOS-STAT3-GAPDH

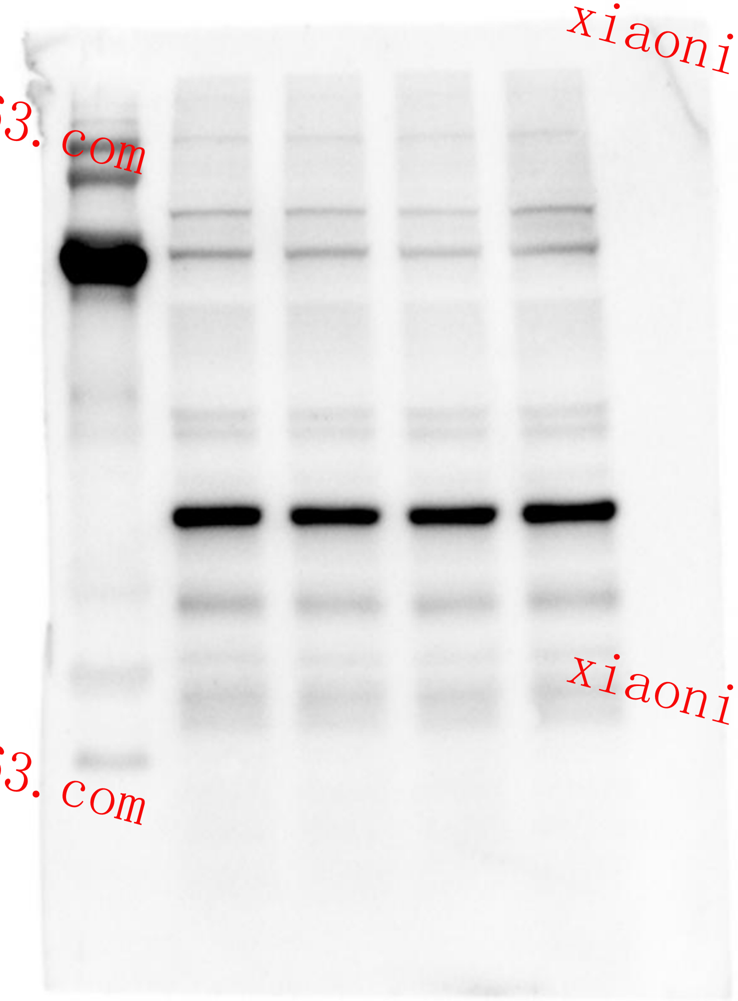

xiaoniu0928@163.com
xiaoniu0928@163.com
xiaoniu0928@163.com
xiaoniu0928@163.com
xiaoniu0928@163.com
xiaoniu0928@163.com

K7M2-STAT3

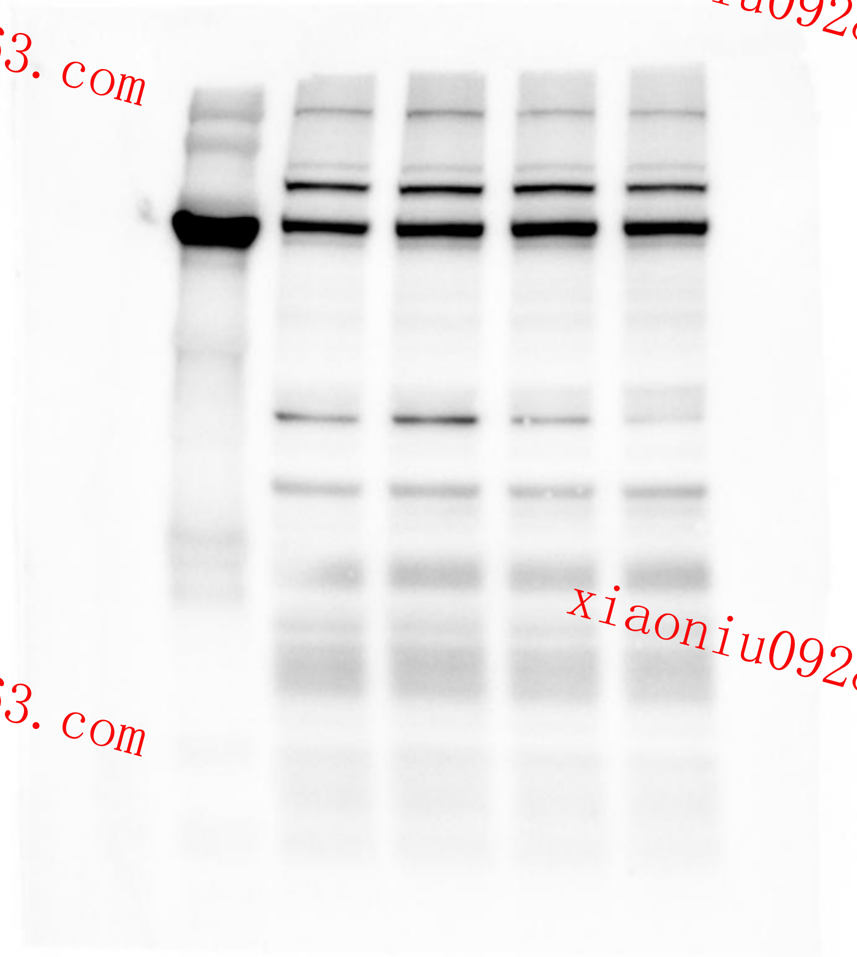

xiaoniu0928@163.com

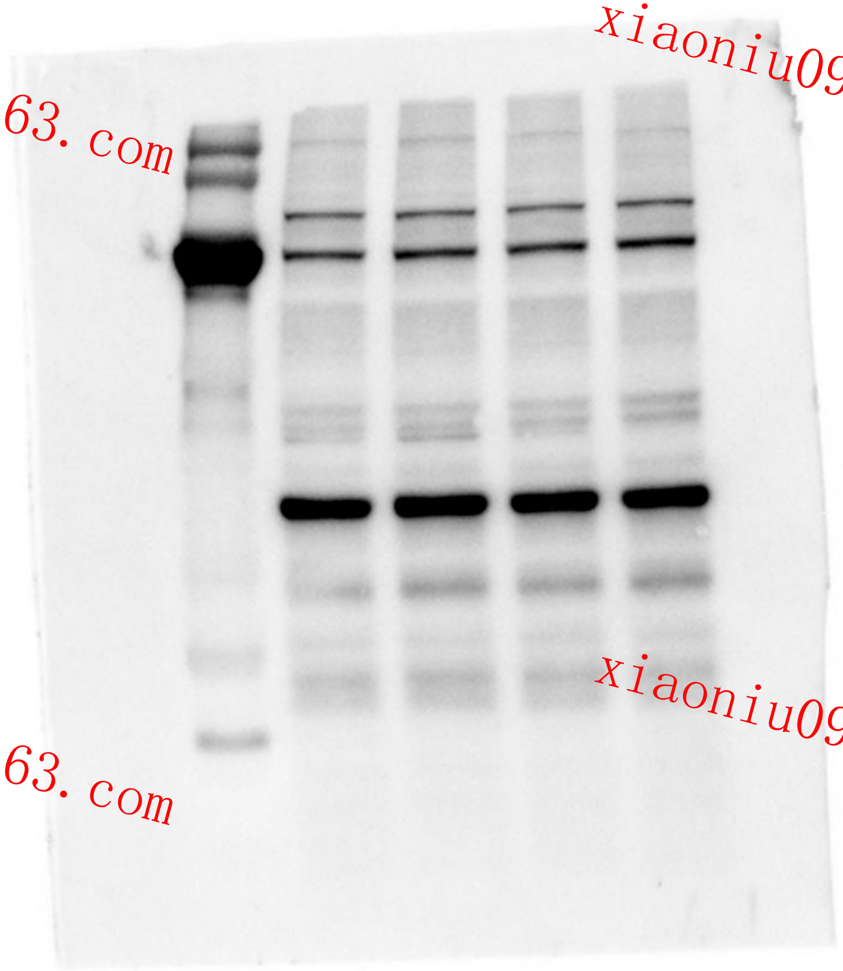

K7M2-STAT3-GAPDH

xiaoniu0928@163.com

HOS-pSTAT3

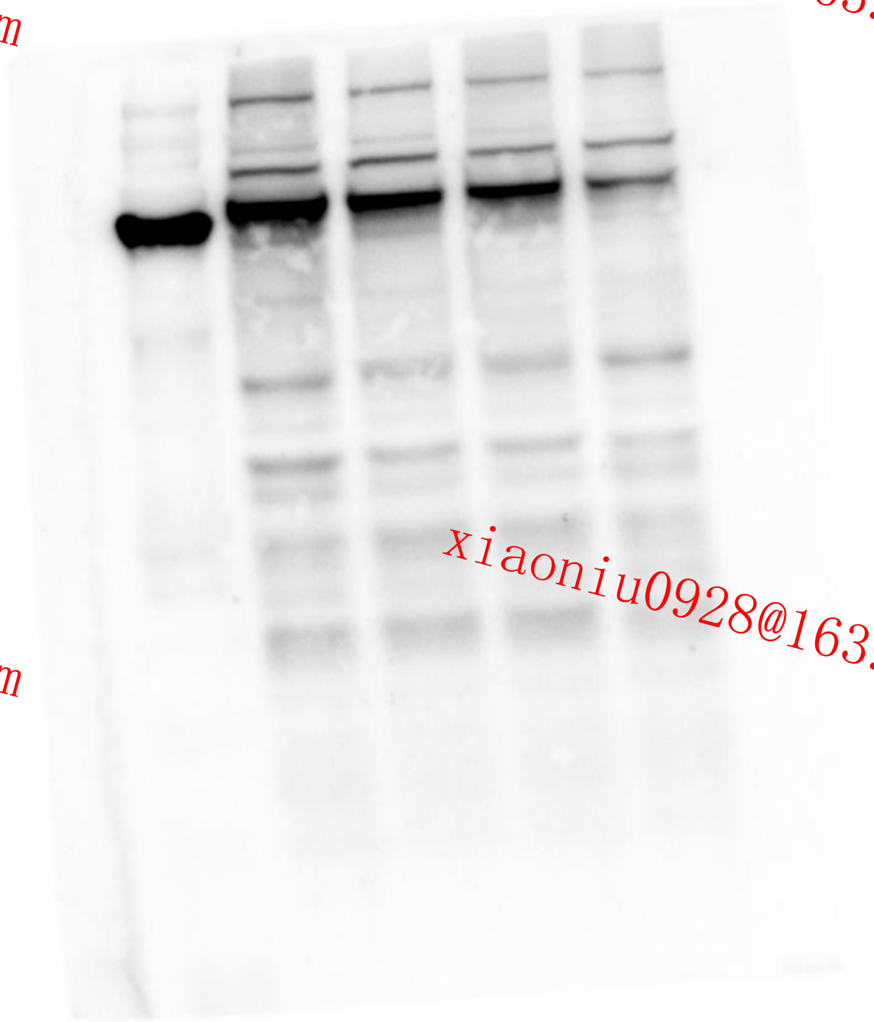

xiaoniu0928@163.com xiaoniu0928@163.com xiaoniu0928@163.com
xiaoniu0928@163.com xiaoniu0928@163.com xiaoniu0928@163.com

HOS-pSTAT3-GAPDH

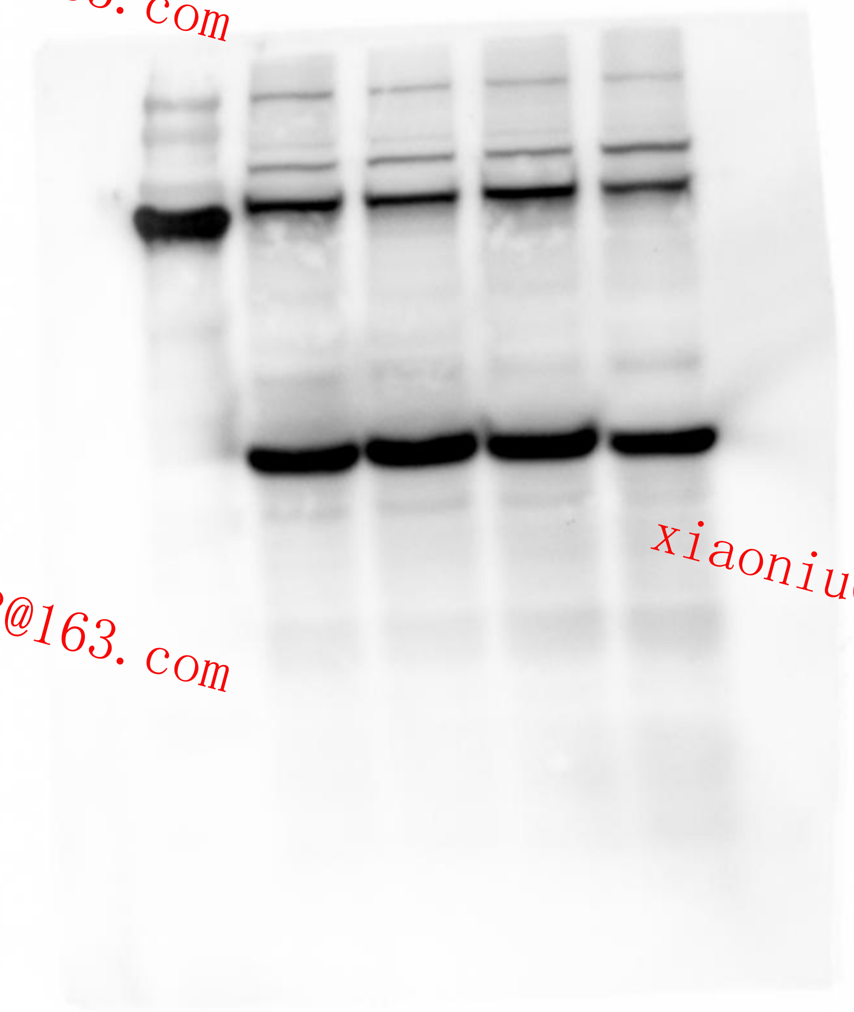

xiaoniu0928@163.com

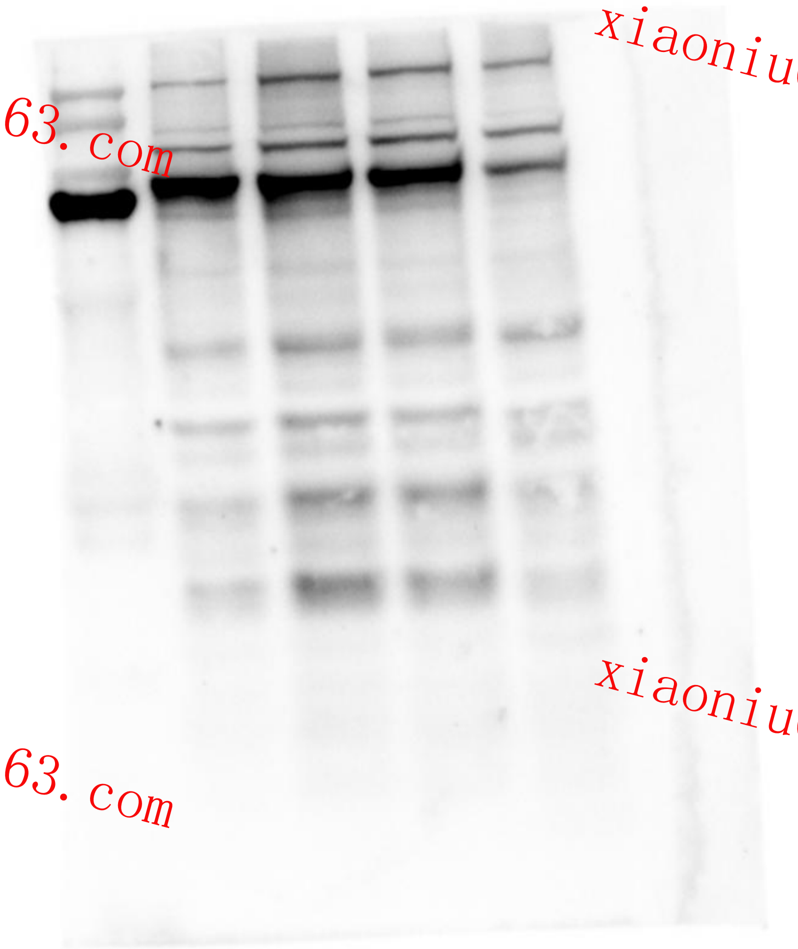

K7M2-pSTAT3

xiaoniu0928@163.com

K7M2-pSTAT3-GAPDH

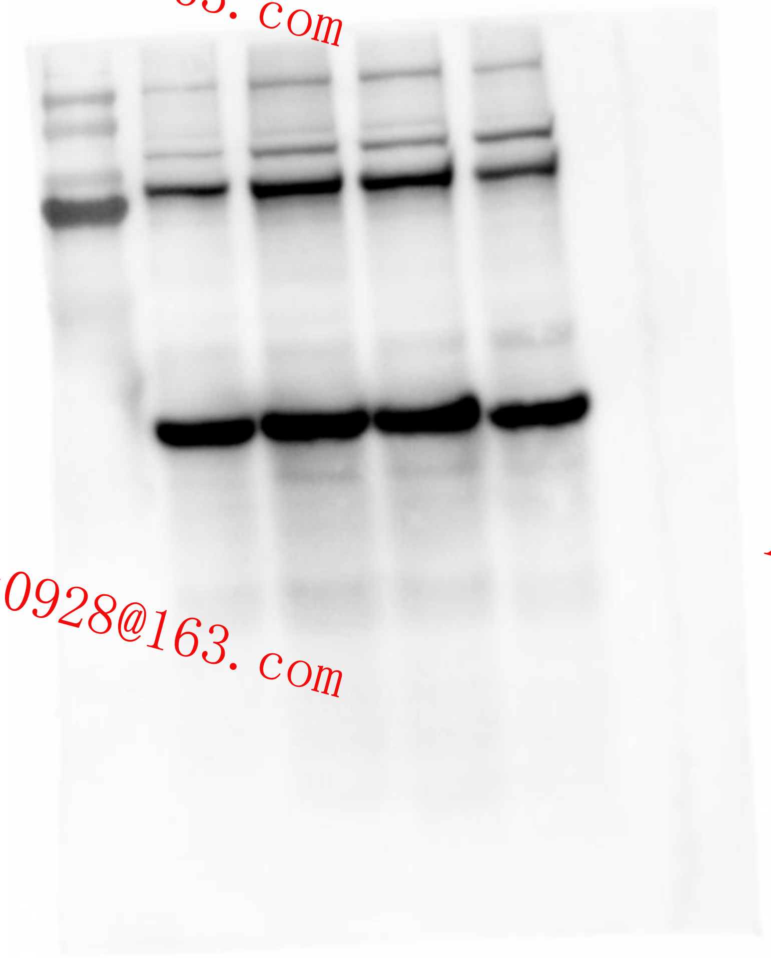

xiaoniu0928@163.com xiaoniu0928@163.com xiaoniu0928@163.com
xiaoniu0928@163.com xiaoniu0928@163.com xiaoniu0928@163.com

Rescue:HOS-PDL1

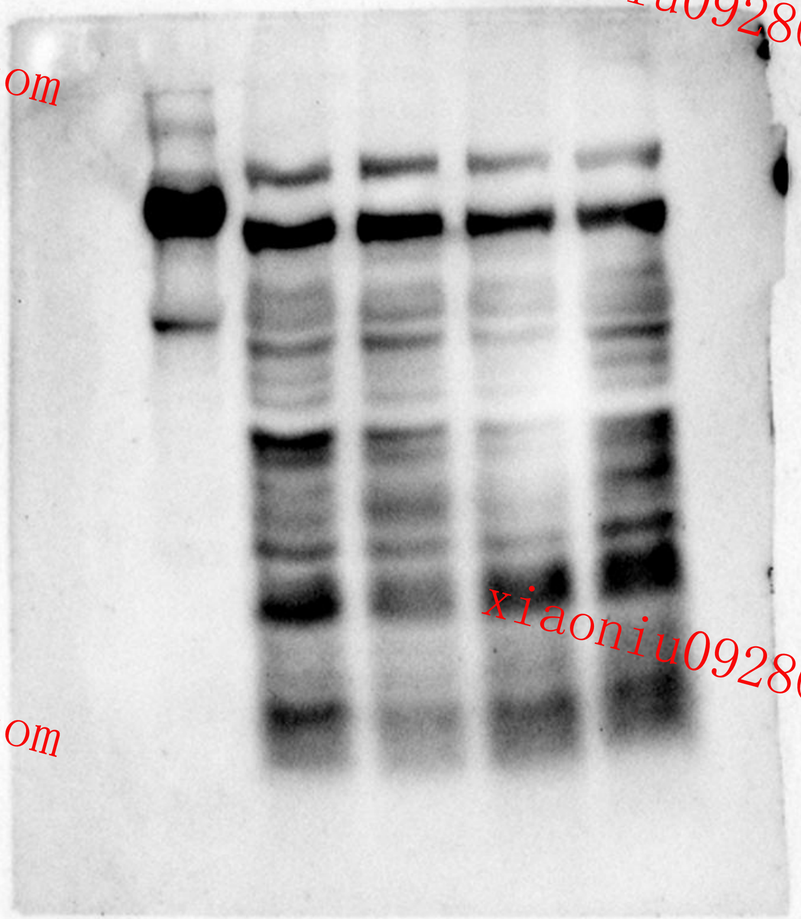

xiaoniu0928@163.com

Rescue:HOS-PDL1-GAPDH

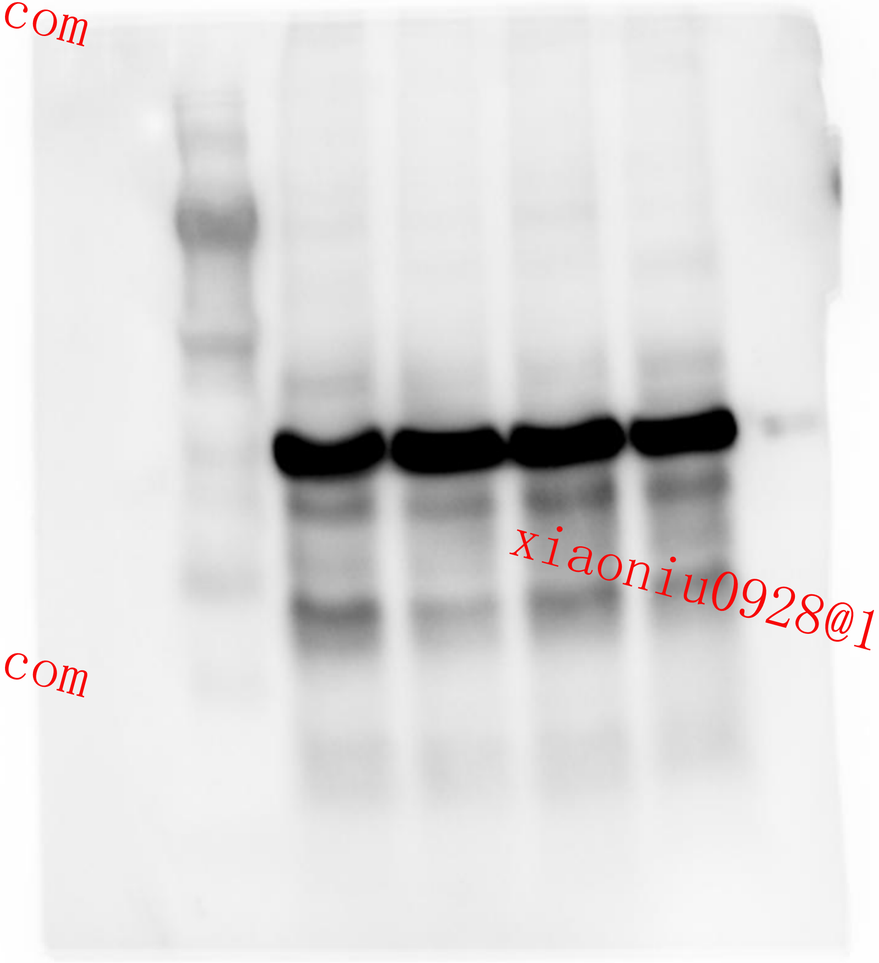

xiaoniu0928@163.com
xiaoniu0928@163.com
xiaoniu0928@163.com
xiaoniu0928@163.com
xiaoniu0928@163.com
xiaoniu0928@163.com

Rescue:HOS-STAT3

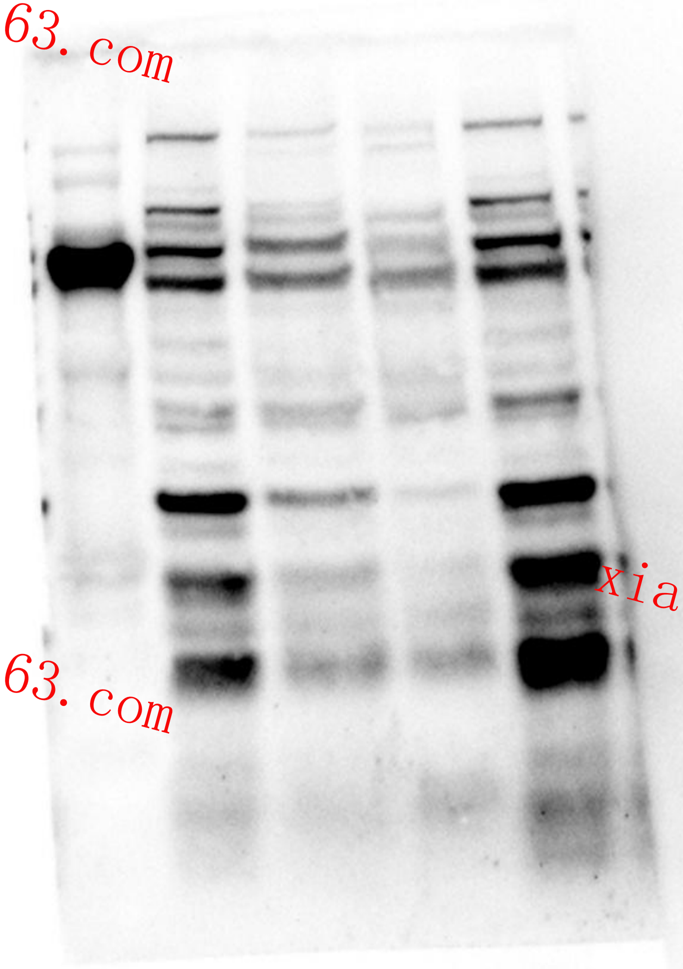

xiaoniu0928@163.com xiaoniu0928@163.com xiaoniu0928@163.com
xiaoniu0928@163.com xiaoniu0928@163.com xiaoniu0928@163.com

Rescue:HOS-STAT3-GAPDH

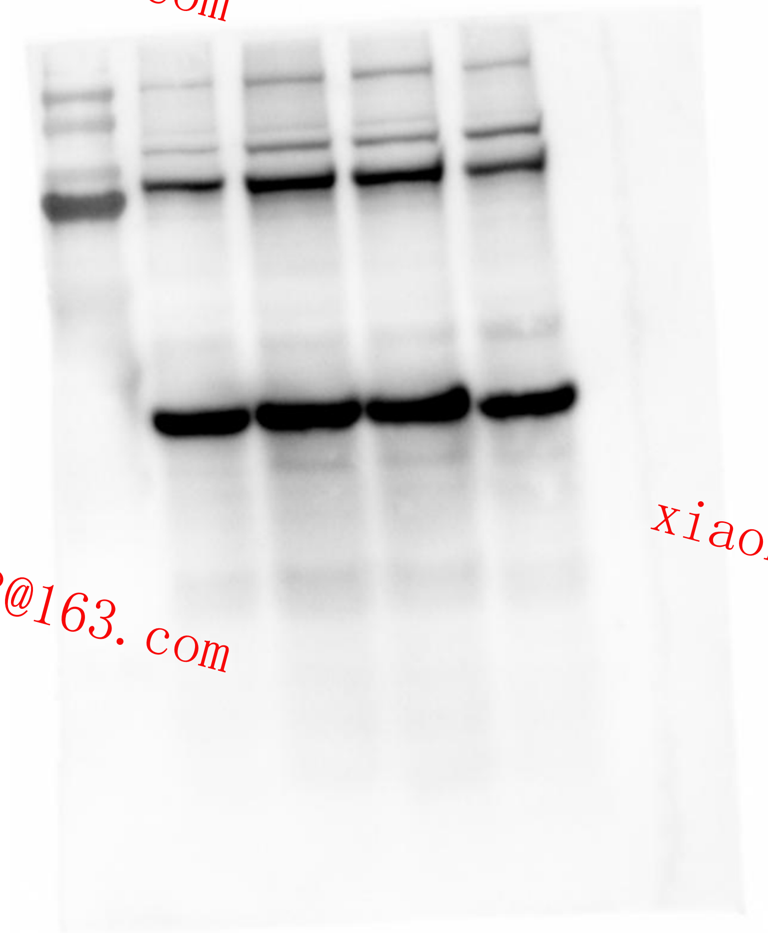

xiaoniu0928@163.com xiaoniu0928@163.com xiaoniu0928@163.com
xiaoniu0928@163.com xiaoniu0928@163.com xiaoniu0928@163.com
