# Supplementary material for: Allicin inhibits PD-L1 through the IL-6/JAK2/STAT3 pathway to suppress immune evasion in osteosarcoma
Source: Front Immunol. 2026 Feb 20;17:1735090. doi: 10.3389/fimmu.2026.1735090 (PMC12962910; doi:10.3389/fimmu.2026.1735090)
Supplement: Supplementary file 2 [file DataSheet2.pdf]

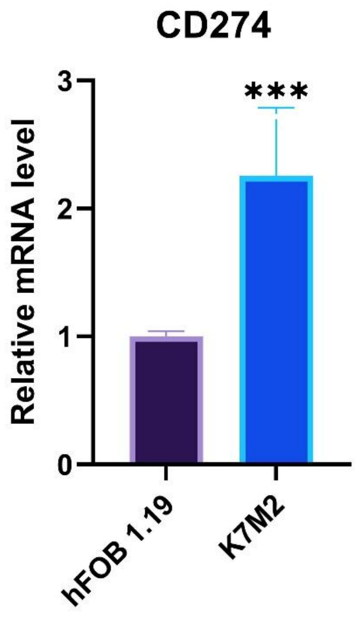

A

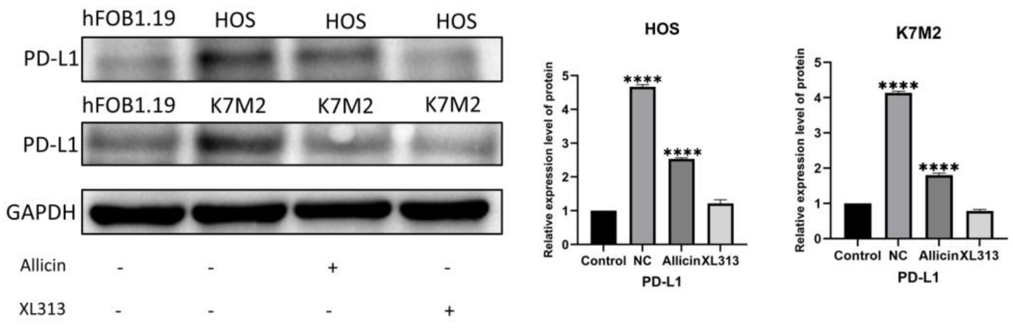

B

Fig. S1 Comparison of CD274 gene expression levels between hFOB1.19 and K7M2 cell lines using RT-PCR (A). Western blot analysis of PD-L1 protein expression in 50 μmol allicin-treated HOS and K7M2 cell lines versus control hFOB1.19 cells, no-treatment controls, and the anti-PD-L1 drug group treated with Mouse denatured collagen type-I Antibody (XL313) group (B).

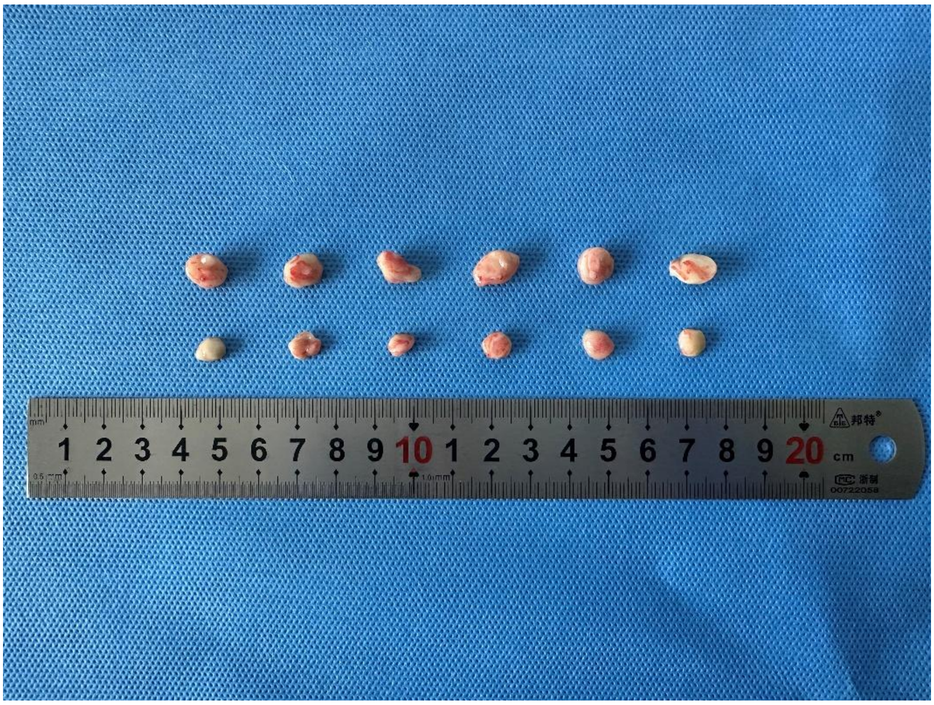

Fig. S2 Images of osteosarcoma samples: top row shows the control group, bottom row shows the allicin-treated group.
