# Supplementary material for: Allicin inhibits PD-L1 through the IL-6/JAK2/STAT3 pathway to suppress immune evasion in osteosarcoma
Source: Front Immunol. 2026 Feb 20;17:1735090. doi: 10.3389/fimmu.2026.1735090 (PMC12962910; doi:10.3389/fimmu.2026.1735090)

Supplement: hFOB1.19 HOS-PDL1

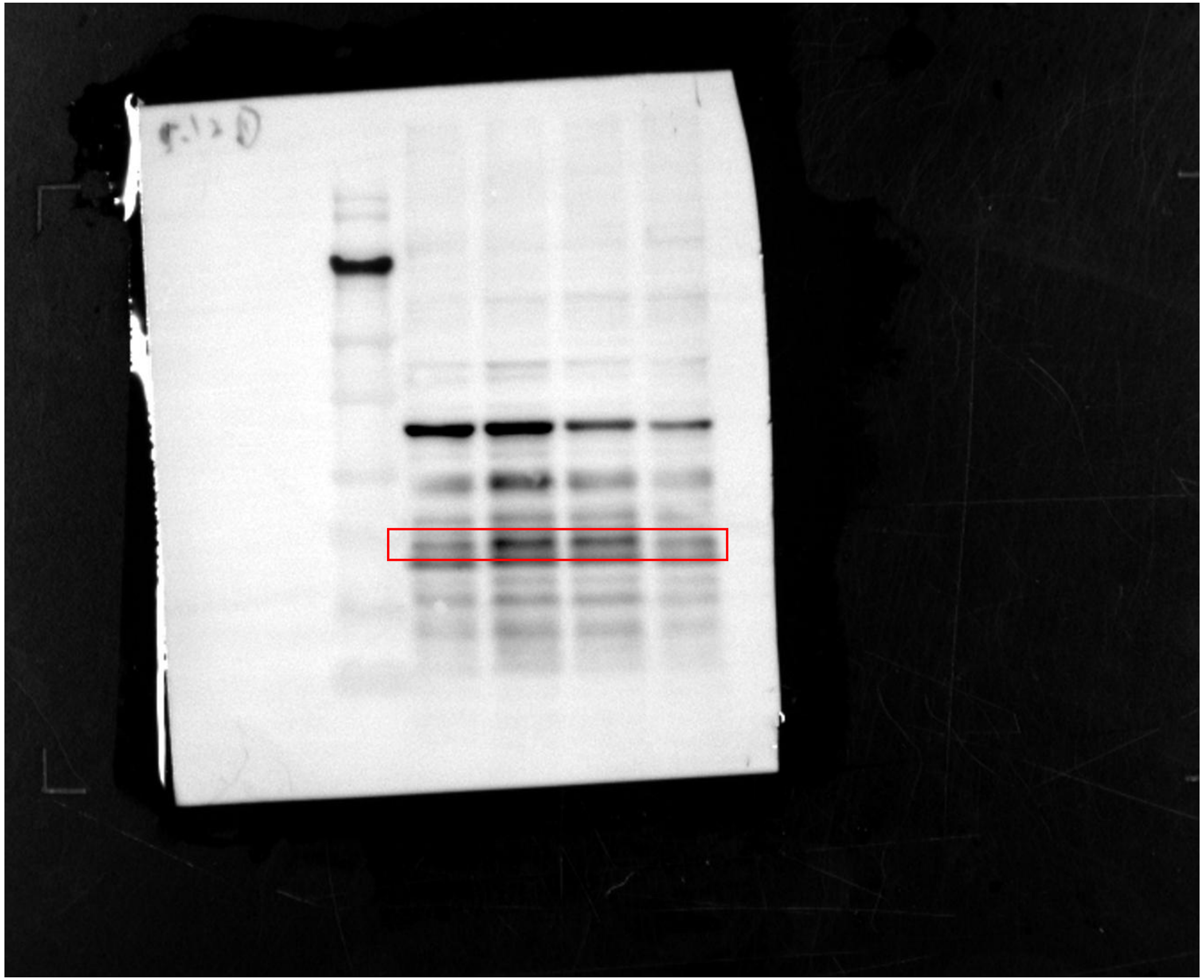

Supplement: hFOB1.19 HOS-PDL1-GAPDH

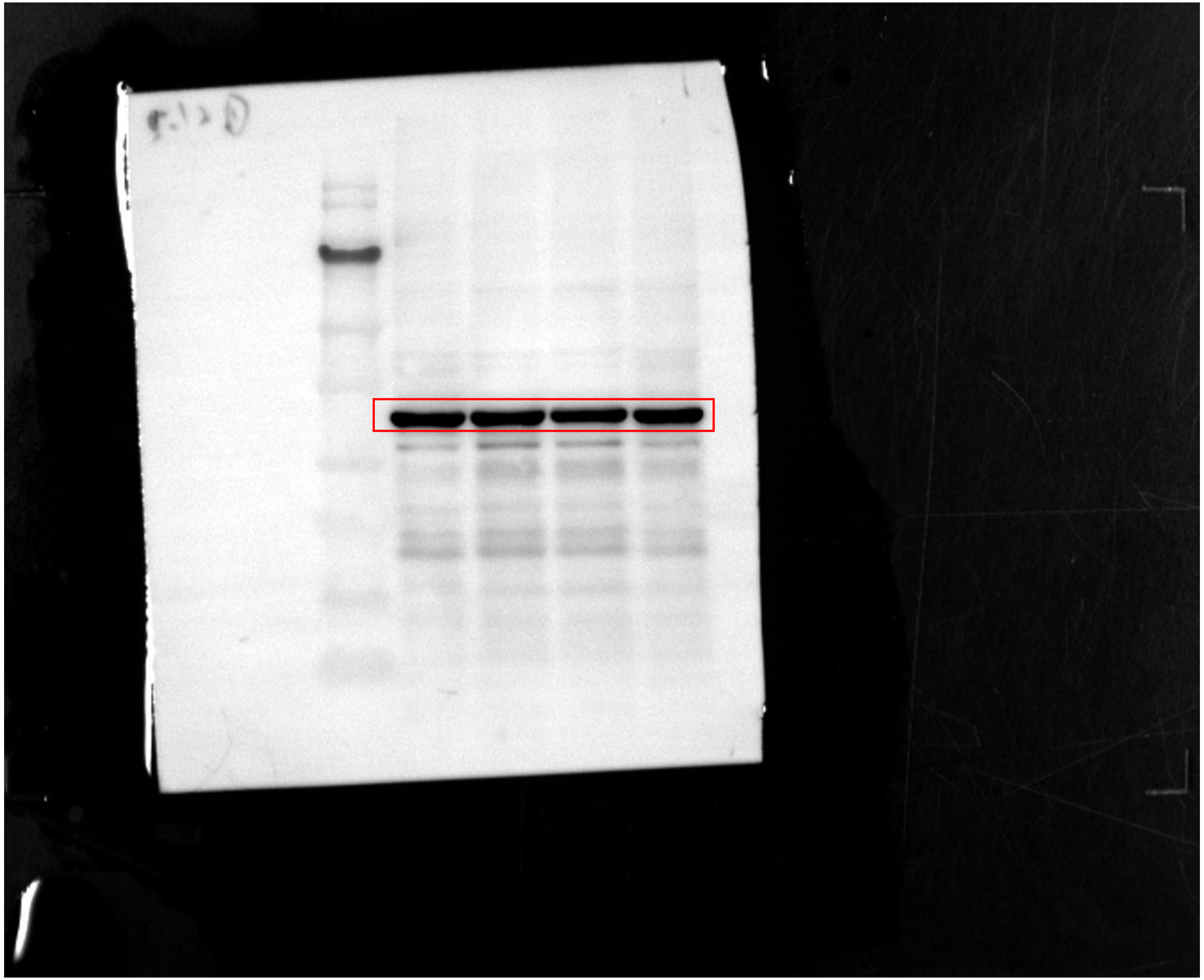

Supplement: hFOB1.19 K7M2-PDL1

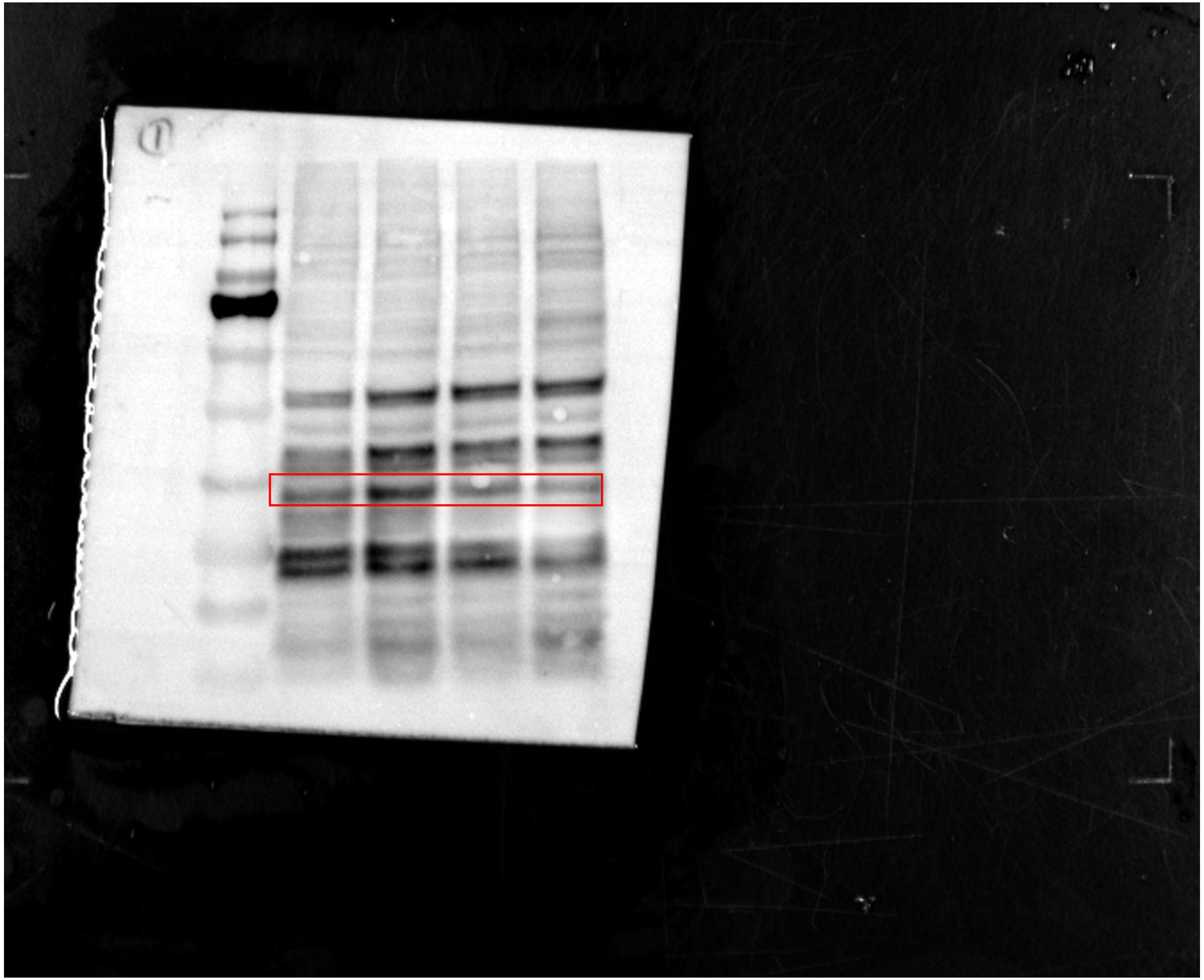

Supplement: hFOB1.19 K7M2-PDL1-GAPDH

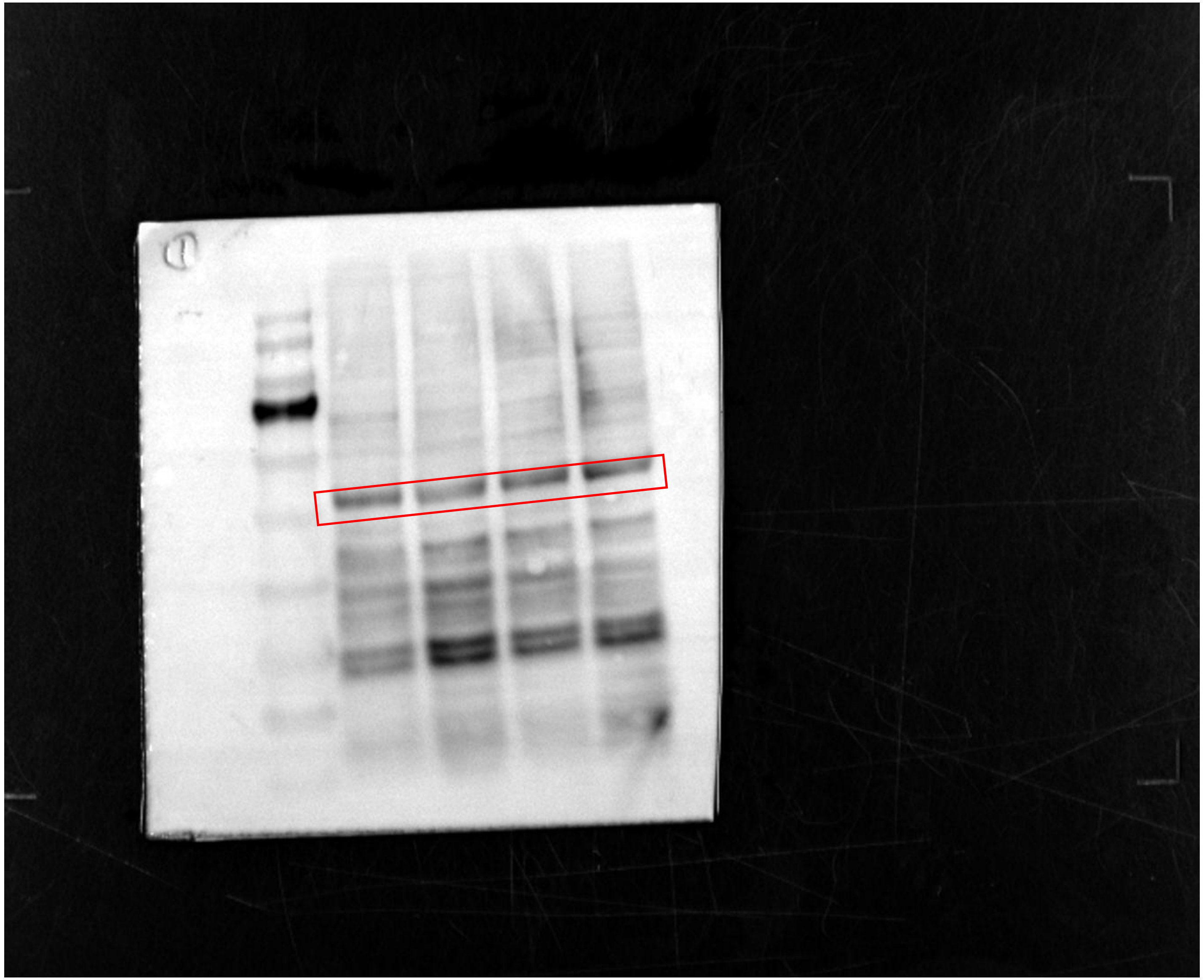

Supplement: Supplementary file 3 [file DataSheet3.pdf]
